# Occupational characteristics are missing from heat vulnerability indices: a study in New York and New Jersey

**DOI:** 10.1186/s12940-026-01284-w

**Published:** 2026-03-14

**Authors:** Zoey Laskaris, Sherry Baron, Steven B. Markowitz

**Affiliations:** Barry Commoner Center for Health & the Environment, Queens College, City University of New York, 65-30 Kissena Blvd, Flushing, NY 11367 USA

**Keywords:** Climate change, Extreme heat, Occupational exposures, Environmental exposures, Social determinants of health, Climate change vulnerability, Outdoor workers

## Abstract

**Background:**

Heat vulnerability indices and maps are widely used by government agencies to guide heat-prevention strategies and prioritize resources for at-risk communities. However, these tools typically exclude the occupational characteristics of the population, a salient source of heat exposure.

**Methods:**

To examine how occupation might enrich these tools, we developed a method to generate Census tract-level estimates of occupational heat exposure risk for employed residents using publicly available data from the U.S. Bureau of Labor Statistics Occupational Information Network (O*NET) and the U.S. Census American Community Survey Public Use Microdata Sample (2018–2022) and applied it to New York State (NYS) and New Jersey (NJ). We calculated the proportion of residents in occupations with elevated indoor or outdoor heat exposure. We then examined correlations and spatial alignment between occupational heat risk and social, health, and environmental vulnerability indicators.

**Results:**

Approximately 1.3 million residents in NYS (13.6% of total employment) and 698,000 in NJ (15.1%) were employed in occupations with elevated indoor or outdoor heat exposure risk. Occupational heat exposure correlated most strongly with health vulnerability, followed by social vulnerability, while environmental vulnerability showed weak or negative correlations. Geographic overlap of tracts in the highest quintiles of occupational risk and other vulnerability domains was modest overall: occupational and health vulnerabilities aligned in less urban areas, whereas occupational, social, and environmental vulnerabilities clustered in and around urban centers.

**Conclusions:**

Integrating occupation into climate and social vulnerability indices is important, because it is an essential human activity, a social determinant of health, and a salient source of heat exposure that is not fully captured by existing social, health, and environmental data. These findings provide a replicable method for mapping occupational heat exposure, highlighting populations at elevated risk, informing future research, and supporting targeted interventions in community and workplace settings, especially where enforceable workplace protections against extreme heat are limited.

**Supplementary Information:**

The online version contains supplementary material available at 10.1186/s12940-026-01284-w.

## Introduction

Climate change-driven increases in the frequency, intensity, and duration of heat waves pose a growing threat to human health [1–3] In response, public health, disaster response, and environmental agencies increasingly rely on heat vulnerability maps to guide risk communication, plan mitigation efforts, and target resources to at-risk communities. Despite being a well-recognized but under-addressed social determinant of health and direct source of heat exposure, [4–8] occupational characteristics of a community (e.g., percent of employed residents in heat-intensive jobs) remain largely absent from existing heat vulnerability indices, including those endorsed by the United States (US) Federal Emergency Management Agency (FEMA), [9, 10] the Centers for Disease Control and Prevention (CDC), [11, 12] the Environmental Protection Agency (EPA), [13] the White House, [14] academic institutions, [15] and state [16] and local governments. [17].

Most existing heat relevant frameworks draw from a conceptual model that characterizes vulnerability through three components: exposure (e.g., surface temperature), susceptibility (e.g., age, income, preexisting health conditions), and adaptive capacity (e.g., access to cooling centers) [18–20] Among these three, susceptibility has received the most attention, reflecting both well-established epidemiologic associations between demographic and socioeconomic factors (e.g., age, income, education, race and ethnicity) and heat-related morbidity and mortality, as well as their role in long-standing health inequities [21–25].

Despite the conspicuous contribution of heat to worker health, [26] occupational characteristics remain largely absent from existing vulnerability frameworks. The U.S. Occupational Safety and Health Administration (OSHA) estimates that occupational heat exposure causes nearly 25,000 illnesses and 559 fatalities annually in the U.S. alone (excluding indirect injuries) [27] Numerous studies have demonstrated a positive relationship between temperature and occupational injuries and fatalities, [28–32] with one study citing a 19% increase in injury rates for every one degree Celsius rise in average summer state temperatures. [33] Outdoor workers face compounded risks from co-occurring climate-related hazards such as wildfire smoke, pesticides, and vector-borne diseases, though the cumulative effects of these exposures are poorly understood. [7, 8].

Moreover, national climate assessments, government agencies, and the media increasingly recognize workers as a priority population for heat prevention, citing their elevated exposure risks, projected economic losses from heat-related work disruptions, and the role of occupational heat as a threat multiplier that can exacerbate heat-related morbidity and mortality among already marginalized populations [1, 2, 34–40].

Thus, excluding occupational characteristics from vulnerability maps may reduce their effectiveness by overlooking an important dimension of risk.

This omission likely reflects multiple factors: a perception that occupational heat exposure falls solely under the jurisdiction of occupational safety agencies, despite the absence of a federal heat standard and state-level protections in only seven states (California, Washington, Minnesota, Oregon, Colorado, Maryland, Nevada); [41] the assumption that income and educational attainment are sufficient proxies for occupational risk; and the lack of high-resolution, detailed occupational data and guidance for integrating it into public health planning, a challenge highlighted during the COVID-19 pandemic [42].

Recognizing these limitations, we developed a method to estimate occupational heat exposure risk at the census tract level in New York State (NYS) and New Jersey (NJ). We quantified the proportion and number of employed residents in heat exposed occupations, mapped geographic variation in risk, and examined how these patterns overlapped with social, health, and environmental vulnerability factors commonly used in existing vulnerability maps. The objective is to determine whether occupational characteristics are already captured within current heat vulnerability data and, if not, to evaluate their value for inclusion in vulnerability indices. High-resolution, publicly available occupational data are paramount for state and local agencies to strengthen their role in addressing occupational heat risk amid the current lack of an enforceable federal standard and limited state-level workplace protections. These methods are replicable in other states, and use of the data can enhance understanding of cumulative risk, guide targeted interventions and public health planning at community and workplace levels, strengthen policy arguments for workplace heat protections, and shape future research directions.

## Methods

We focused our analysis on NYS and NJ, two economically diverse sizable Northeastern states experiencing increases in the frequency and duration of multi-day heat waves and representing policy-relevant settings where worker heat protection legislation has not yet been enacted. [43, 44] We utilized census tracts as the primary unit of analysis. Tracts were selected due to the availability of relevant heat vulnerability data at this geographic level in the social, health, and environmental domains and their public health significance. Table 1 lists the sources and details of the data utilized in this study.

**Table 1:** Data sources and details

Data Source (Year)	Abbreviation	Variables	Vulnerability Domain	Geography
U.S. Bureau of Labor Statistics Occupational Information Network (2025 release, version 29.2)	BLS O*NET	• Frequency of working “outdoors, exposed to weather” or “outdoors, under cover” • Frequency of working “indoors, in an environmentally uncontrolled environment”	Occupational Heat Exposure	*Not applicable*
U.S. American Community Survey (5-year, 2018–2022)	ACS	• Major Occupation Group (2-digit SOC code) • Employed civilian labor force (16 + years)	Occupational Heat Exposure	Tract (Small administrative units (1,200–8,000 people). Provides representative group-level estimates for all U.S. residential areas
U.S. American Community Survey Public Use Microdata Sample (5-year, 2018–2022)	PUMS	• Detailed Occupation (4–6-digit SOC code)	Occupational Heat Exposure	Public Use Microdata Areas (PUMAs) Large statistical areas (min. 100,000 people). Provides a representative 5% sample of individual-level U.S. population records
U.S. Environmental Protection Agency Environmental Justice Screening Tool (2024 release, with sociodemographic indicators derived from the ACS 2018–2022 5-year estimates and air quality indicators based on 2020 modeled or monitored source data)	EPA EJSCREEN	• People of color – Percent • Low income – Percent • Less than High School Education—Percent • Limited English-speaking households- Percent • Individuals Over Age 64 • Percent Particulate Matter 2.5 (μg/m3) • Ozone (PPM) • Nitrogen Dioxide (PPB)	Social Determinants & Environmental Risk Factors	Tract
U.S. Federal Emergency Management Agency National Risk Index (2023 release, with heat wave frequency estimates derived from National Weather Service data spanning 2005–2024)	FEMA NRI	• Heat Wave—Annualized Frequency	Environmental Risk Factors	Tract
U.S. Centers for Disease Control PLACES (2022 release, using Behavioral Risk Factor Surveillance System (BRFSS) data from 2019–2020)	CDC PLACES	• Coronary Heart Disease among Adults—Crude Prevalence • Chronic Obstructive Pulmonary Disease among Adults—Crude Prevalence • Current Asthma among Adults—Crude Prevalence • Diabetes among Adults—Crude Prevalence • High Blood Pressure among Adults—Crude Prevalence	Health Risk Factors	Tract
Integrated Public Use Microdata Series National Historical Geographic Information System (2020 Decennial Census Demographic and Housing Characteristics)	IPUMS NHGIS	• Total and urban population • GIS 2022 TIGER/Line files for 2020 census tract boundaries	*Not applicable*	Tract

### Occupational heat exposure

Several steps were involved in estimating the percentage of employed residents at risk of occupational heat exposure by tract. The first step was to describe the risk of both outdoor and indoor heat exposure across various occupations. To do this for U.S. based occupations, we used data from the U.S. Bureau of Labor Statistics (BLS) Occupational Information Network (O*NET, version 29.2). [45] O*NET is a publicly accessible database that provides detailed information about the characteristics of 923 unique occupations, with data collected through surveys completed by both workers and occupational experts and periodically updated. We focused on heat exposure risk in both outdoor and indoor environments. Indoor workspaces without environmental controls are affected by external temperatures, and several existing and proposed workplace heat protections explicitly include indoor workers (e.g., Minnesota, California, Maryland). [41] Radiant heat, such as from furnaces or stoves, are not uniquely classified in O*NET and were therefore excluded from this analysis.

To describe the risk level of working in outdoor heat, we analyzed responses to two questions from the O*NET Work Context survey: “How often does your current job require you to work outdoors, exposed to all weather conditions?” and “How often does your current job require you to work outdoors, under cover (like in an open shed)?” [45] To describe the risk level of indoor heat exposure, we used the question “How often does your current job require you to work indoor in an environment that is not environmentally controlled (like a warehouse without air conditioning)?” [45] These questions used a 5-point frequency scale that was standardized to a 0–100 metric for interpretability, where 0 = never, 25 = once a year or more but not every month, 50 = once a month or more but not every week, 75 = once a week or more but not every day, and 100 = every day.

We converted O*NET occupation codes with non-missing data (*n* = 879) into 2018 Standard Occupational Classification (SOC) codes using a crosswalk file provided by the ONET Resource Center. [46] For cases where multiple O*NET codes corresponded to a single SOC code, we imputed the mean heat exposure scores for those SOC classifications. The final output was a list of 763 SOC codes along with their corresponding indoor and outdoor heat risk scores.

#### Definition of heat exposed occupations

To identify occupations *at risk* of indoor or outdoor heat exposure (henceforth, heat exposed occupations) we used a threshold approach. Occupations with heat risk scores of 75 or higher on either of the outdoor or indoor heat characteristics were considered at risk of occupational heat exposure. A score of 75 is the equivalent of being exposed once a week or more but not every day.

Because heat-related physiological and cognitive effects can occur within a single shift, we focused on occupations with at least weekly exposure; including those with only monthly or rarer exposure could overestimate meaningful occupational risk. This definition aligns with occupational heat prevention standards that protect workers exposed to heat for more than brief periods (≥ 15 min) once temperatures exceed established thresholds.

#### Number and percentage of employed residents exposed to occupational heat by census tract

To calculate the number and percentage of the civilian employed population (16 + years of age) residing in each census tract in NYS and NJ who are at risk of occupational heat exposure, we accessed employment data from the 2018–2022 U.S. American Community Survey (ACS) using the tidy census R package. [47, 48].

We retrieved employment estimates at both the tract and Public Use Microdata Area (PUMA) geographic levels. [49] In the U.S. Census hierarchy, census tracts are small subdivisions (1,200–8,000 residents); to protect confidentiality in these small populations, occupational data are released only in aggregated form and are limited to broad, 2-digit “major” occupation group SOC codes. [50] In contrast, PUMAs are larger areas (min. 100,000 residents) that provide a representative 5% sample of de-identified individual-level records—the Public Use Microdata Sample (PUMS)—with more detailed 4–6 digit PUMS-specific SOC (SOCP) codes. [49].

Because tract-level ACS data are restricted to major occupation groups that can mask substantial within-group variation in heat exposure, we used detailed PUMS occupations to classify heat-exposed work and to generate PUMA-level occupational distributions that were then applied to tracts, minimizing exposure misclassification. For example, within the Construction and Extraction major group (47–0000), pipelayers have an outdoor heat exposure risk score of 100.0, while carpet, floor, and tile installers have a score of 28.0. Leveraging these detailed PUMS data allowed us to capture these internal variations and improve the accuracy of our tract-level risk estimates using the best available ACS data. Specifically, we first estimated the proportion of workers in each major occupation group who were heat-exposed within each PUMA based on detailed occupational titles and then applied these proportions to tract-level major occupation group counts to generate tract-specific exposure estimates.

We linked 763 SOC codes to 531 SOCP codes (*n* = 531) using ACS technical documentation. [51] Because the Census Bureau aggregates certain SOC occupations into single SOCP categories for public-use data, 59 SOC codes mapped to a single SOCP. For example, specific roles such as Janitors (37–2011), Maids and Housekeeping Cleaners (37–2012), and all other Building Cleaners (37–2019) were collapsed into the single SOCP code 37-201X. For these aggregated occupations, we calculated the mean and median ONET-derived heat exposure scores and retained the higher values; on average, the measures differed by < 0.5 points on a 0–100 scale, indicating minimal impact on exposure classification while reducing potential underestimation. After excluding SOCP codes with missing ONET data, including military occupations, the “unemployed, with no work experience in the past 5 years”, and records with missing occupation, we retained 499 unique SOCP codes with corresponding heat exposure risk scores.

Using the 2018–2022 ACS PUMS data, we estimated the number of employed residents within each PUMA at risk of occupational heat exposure by summing person-level sampling weights (“person-weights”) by detailed occupation. [51] To align geographic boundaries across census years, an allocation factor for converting between 2010 and 2020 PUMA geographies was obtained from the GEOCORR geographic correspondence engine developed by the Missouri Census Data Center. [52] These weighted totals were then used to generate, within each PUMA, the percentage of workers in each major occupation group who were heat-exposed based on detailed occupational titles. Because census tracts nest entirely within PUMAs, we applied these exposure proportions to tract-level major occupation group employment counts, assuming that the occupational composition of tract residents reflects that of their parent PUMA. This yielded tract-level estimates of the number of employed residents at risk of occupational heat exposure across NY and NJ.

### Selection of social, health, and environmental heat vulnerability factors

To explore the relationship between our derived occupational heat exposure variable and commonly used indicators of heat vulnerability, we accessed publicly available tract-level data (see Table 1). Rather than applying our measure to a composite index, we examined its association with a targeted set of social, health, and environmental variables. Indicators were selected based on prior inclusion in social and heat vulnerability indices, established associations with heat-related morbidity and mortality, relevance to heat vulnerability research conducted in the Northeastern United States, and availability at the census tract level. [16, 23, 25, 53–55] Examining indicators individually improves interpretability by allowing identification of the specific factors driving spatial alignment with occupational heat exposure relationships that may be obscured when variables are aggregated into a composite score. [56] Associations between occupational heat exposure and each indicator were assessed using pairwise Spearman rank correlations, supplemented by visual comparison of tract-level spatial distributions. This analysis was exploratory and hypothesis-generating, with the objective of determining whether occupational heat exposure represents a distinct spatial dimension of vulnerability not already captured by commonly used indicators and evaluating its potential value for future vulnerability indices.

#### Social determinants

Social factors such as age, race, ethnicity, English-language ability, educational attainment, and income can influence heat exposure, susceptibility, and adaptive capacity through both individual- and community-level pathways. [22, 23, 25, 57, 58] These pathways include housing and neighborhood conditions, heat risk perception, access to private air conditioning or cooling centers, and use of medication that may affect thermoregulation (e.g., insulin treatment to treat diabetes). [19, 55, 57, 59] Tract-level data for these factors were obtained from the U.S. EPA Environmental Justice Screening and Mapping Tool (EJSCREEN, 2024 release, using ACS 2018–2022 5-year summary data) [13].

#### Health risk factors

Populations with preexisting health conditions, such as heart disease, diabetes, and pulmonary conditions, are more susceptible to heat-related illnesses. [55, 60–62] To capture this dimension of vulnerability, we included tract-level data on the prevalence of coronary heart disease (CHD), chronic obstructive pulmonary disease (COPD), asthma, diabetes, and high blood pressure (HBP) among adults (Table 1). These data were obtained from the U.S. Centers for Disease Control (CDC) PLACES database (2022 release, using Behavioral Risk Factor Surveillance System (BRFSS) data from 2019–2020). [63].

#### Environmental risk factors

While numerous natural and built-environmental features influence land surface temperatures and human exposure to heat, we focused in this study on two environmental risk factors particularly relevant to heat-health interactions: (1) the historical frequency of heat events and (2) air quality.

We obtained tract–level data from the Federal Emergency Management Agency (FEMA, 2023 release), which incorporates heat wave frequency estimates derived from National Weather Service observations spanning 2005–2024, to characterize historical exposure to extreme heat measured as the annual frequency of heat waves. [10] These data served as a proxy for the local intensity and regularity of extreme heat across the study area.

Air quality was included as a second environmental risk factor due to its documented interaction with heat in affecting adverse health outcomes. [64–68] Poor air quality, including elevated levels of ozone and fine particulate matter (PM_2.5_), often coincides with high temperatures and can amplify the risk of heat-related illness by compounding stress on the thermoregulatory, pulmonary, and cardiovascular systems. [69, 70] This is particularly relevant for outdoor workers, who may face compounded risks from both heat and air pollution. [7] Annual mean measures of PM_2.5_, ozone, and Nitrogen Dioxide (NO_2_) were obtained at the tract level from the U.S. EPA’s EJSCREEN tool (2024 release, with air quality indicators derived primarily from 2020 source data). [13].

To account for local land use and urban design features that influence heat exposure (e.g., green space, impervious surfaces, and population density), [23, 54] we stratified our analyses by urbanicity. Urbanicity was calculated using 2020 Decennial Census data from the Integrated Public Use Microdata Series National Historical Geographic Information System (IPUMS NHGIS) and defined as the percentage of a tract's population residing in urban-designated areas. [71] Tracts were classified as urban (≥ 75% urban population) or non-urban (< 75%). To validate this classification, we cross-referenced our results using data from the National Land Cover Database (NLCD), accessed at the tract-level via the National Neighborhood Data Archive (NaNDA). [72] As expected, urban tracts exhibited more impervious surfaces and less vegetation cover than non-urban tracts (data not shown).

### Geographic data

We used 2022 TIGER/Line Census Tract Boundaries from IPUMS NHGIS to define the geographic units for analysis. [71] These boundaries reflect the most recent tract delineations following the 2020 census and include tract to PUMA relationships in Geographic Information System (GIS) format. We integrated these boundaries with our derived measure of occupational heat exposure risk, as well as external data on social, health, economic and environmental factors, using ArcGIS Pro. [73] A total of 5,257 tracts from NY and 2,160 tracts from NJ were included. Tracts with fewer than 50 employed persons were excluded from this analysis (NY: *n* = 154; NJ: *n* = 21) to reduce instability in percentage estimates driven by small denominators.

### Statistical analysis

We conducted univariate analyses to describe the distribution of indoor and outdoor occupational heat exposure risk scores across major occupation groups. Descriptive statistics were generated for all variables in the occupational, social, health, and environmental vulnerability domains for tracts in NY and NJ, stratified by urbanicity. To assess relationships between occupational heat exposure and other factors, we calculated Spearman correlation coefficients (ρ) at the tract level, stratified by state and urbanicity.

To identify communities experiencing disproportionate vulnerability, we applied percentile-based thresholds, a commonly used method in climate change and heat vulnerability indices. Occupational heat vulnerability was defined as ranking ≥ 80th percentile for the proportion of employed residents exposed to indoor or outdoor heat. The ≥ 80th percentile threshold was selected a priori based on its common use in established vulnerability indices. We conducted sensitivity analyses applying alternative thresholds (≥ 70th and ≥ 90th percentiles) to examine the sensitivity of tract classifications to threshold selection. High social inequity (“social vulnerability”) was defined as ranking ≥ 80th percentile for low-income in combination with at least one of the following: proportion of people of color, population aged 65 +, educational attainment below high school, or limited English proficiency. Health and environmental exposure vulnerabilities were assigned to tracts ranking ≥ 80th percentile for any single indicator within their respective domains.

We then quantified the number of census tracts experiencing an occupational heat vulnerability that also ranked as vulnerable in the social, health, or environmental domains. Geographic patterns of overlapping vulnerabilities were visualized using ArcGIS Pro. [73] All statistical analyses were conducted using RStudio. [74].

## Results

### Occupational heat exposure

#### Heat risk by occupation

Outdoor and indoor occupational heat exposure risk scores for detailed occupations varied widely across Major Occupational Groups (Table 2 shows the top five occupation groups by median risk score with example occupations; Supplemental Table 1 includes all groups; Supplemental Table 2 shows risk scores for all 763 occupations). Major group summaries are presented to illustrate within-group heterogeneity in exposure risk. Using a threshold score of 75 (equivalent to exposure on one or more days per week), 16 major groups included at least one detailed occupation at risk of outdoor heat exposure, and 10 for indoor heat exposure.

**Table 2:** Top five major occupation groups by median outdoor and indoor heat exposure risk scores with exemplar job titles

		**Heat Exposure Risk Score**^**a**^		
**Heat Type**	**Major Occupation Group (SOC Code)**^**b**^	**Median**	**Mean (SD)**	**Range (min, max)**
	*Exemplar occupations with elevated risk of heat exposure (heat exposure risk score)*^*c*^			
**Outdoor Heat (work outdoors, exposed to all weather)**^**d**^				
	**Building and Grounds Cleaning and Maintenance Occupations (37–0000)**	92.9	70.8 (36.7)	7.8, 99.5
	*Landscaping and Groundskeeping Workers (99.5)*			
	*Tree Trimmers and Pruners (97.8)*			
	*Pesticide Handlers, Sprayers, and Applicators, Vegetation (94.0)*			
	**Farming, Fishing, and Forestry Occupations (45–0000)**	84.4	80.9 (22.0)	16.2, 99.0
	*Log Graders and Scalers (99.0)*			
	*Fishing and Hunting Workers (95.2)*			
	*Farmworkers and Laborers, Crop, Nursery, and Greenhouse (86.2)*			
	**Construction and Extraction Occupations (47–0000)**	83.8	75.0 (25.4)	19.0, 100.0
	*Operating Engineers and Other Construction Equipment Operators (100.0)*			
	*Pipelayers (100.0)*			
	*Roofers (99.5)*			
	**Protective Service Occupations (33–0000)**	81.9	67.2 (29.0)	12.5, 99.8
	*Crossing Guards and Flaggers (99.8)*			
	*Parking Enforcement Workers (99.5)*			
	*Fish and Game Wardens (96.8)*			
	**Transportation and Material Moving Occupations (53–0000)**	78.8	71.9 (23.7)	3.2, 100.0
	*Driver/Sales Workers (100.0)*			
	*Refuse and Recyclable Material Collectors (97.2)*			
	*Aircraft Cargo Handling Supervisors (84.8)*			
**Indoor Heat (work indoors without environmental controls)**^**e**^				
	**Installation, Maintenance, and Repair Occupations (49–0000)**	73.1	66.1 (22.0)	0.0, 98.5
	*Wind Turbine Service Technicians (94.2)*			
	*Tire Repairers and Changers (90.8)*			
	*Farm Equipment Mechanics and Service Technicians (90.5)*			
	**Production Occupations (51–0000)**	56.8	51.1 (26.0)	2.0, 99.0
	*Pourers and Casters, Metal (99.0)*			
	*Chemical Plant and System Operators (94.0)*			
	*Chemical Plant and System Operators (88.8)*			
	**Construction and Extraction Occupations (47–0000)**	56.6	56.3 (15.1)	24.0, 89.5
	*Helpers–Extraction Workers (89.5)*			
	*Electricians (80.5)*			
	*Plumbers, Pipefitters, and Steamfitters (76.9)*			
	**Transportation and Material Moving Occupations (53–0000)**	53.0	51.9 (20.6)	6.0, 86.5
	*Cleaners of Vehicles and Equipment (82.0)*			
	*Packers and Packagers, Hand (81.5)*			
	*Industrial Truck and Tractor Operators (80.2)*			
	**Farming, Fishing, and Forestry Occupations (45–0000)**^**f**^	51.4	47.1 (19.9)	16.2, 75.5
	*Forest and Conservation Workers (75.5)*			

^a^Risk score values (0–100) correspond approximately to 0 = Never, 25 = Once a year or more but not every month, 50 = Once a month or more but not every week, 75 = Once a week or more but not every day, and 100 = Every day. ^b^See Supplemental Table 1 for the distribution of heat exposure risk scores for all major occupation groups. ^c^See Supplemental Table 2 for heat exposure risk scores for all detailed occupation titles. ^d^Outdoor heat exposure risk scores were derived from two O*NET Work Context Survey questions: “How often does your current job require you to work outdoors, exposed to all weather conditions?” and “How often does your current job require you to work outdoors, under cover (like in an open shed)?” Responses were on a 5-point frequency scale, converted to 0–100 scale. ^e^Indoor heat exposure risk was based on the O*NET question: “How often does your current job require you to work indoors in an environment that is not environmentally controlled (like a warehouse without air conditioning)?” and scored using the same method. ^f^Only one occupation in Farming, Fishing, and Forestry Occupations had an elevated risk of indoor heat exposure using the risk score threshold of 75 or greater

Outdoor heat risk (i.e., outdoors exposed to weather or under cover) was highest in Building and Grounds Cleaning and Maintenance, Farming, Fishing, and Forestry, Construction and Extraction, Protective Service, and Transportation and Material Moving occupations.

Indoor heat exposure risk (reflecting work in environments lacking climate control) was highest in Installation, Maintenance, and Repair, Production, Construction and Extraction, and Transportation and Material Moving.

#### Occupational heat risk exposure burden in NYS and NJ

**Fig. 1:**
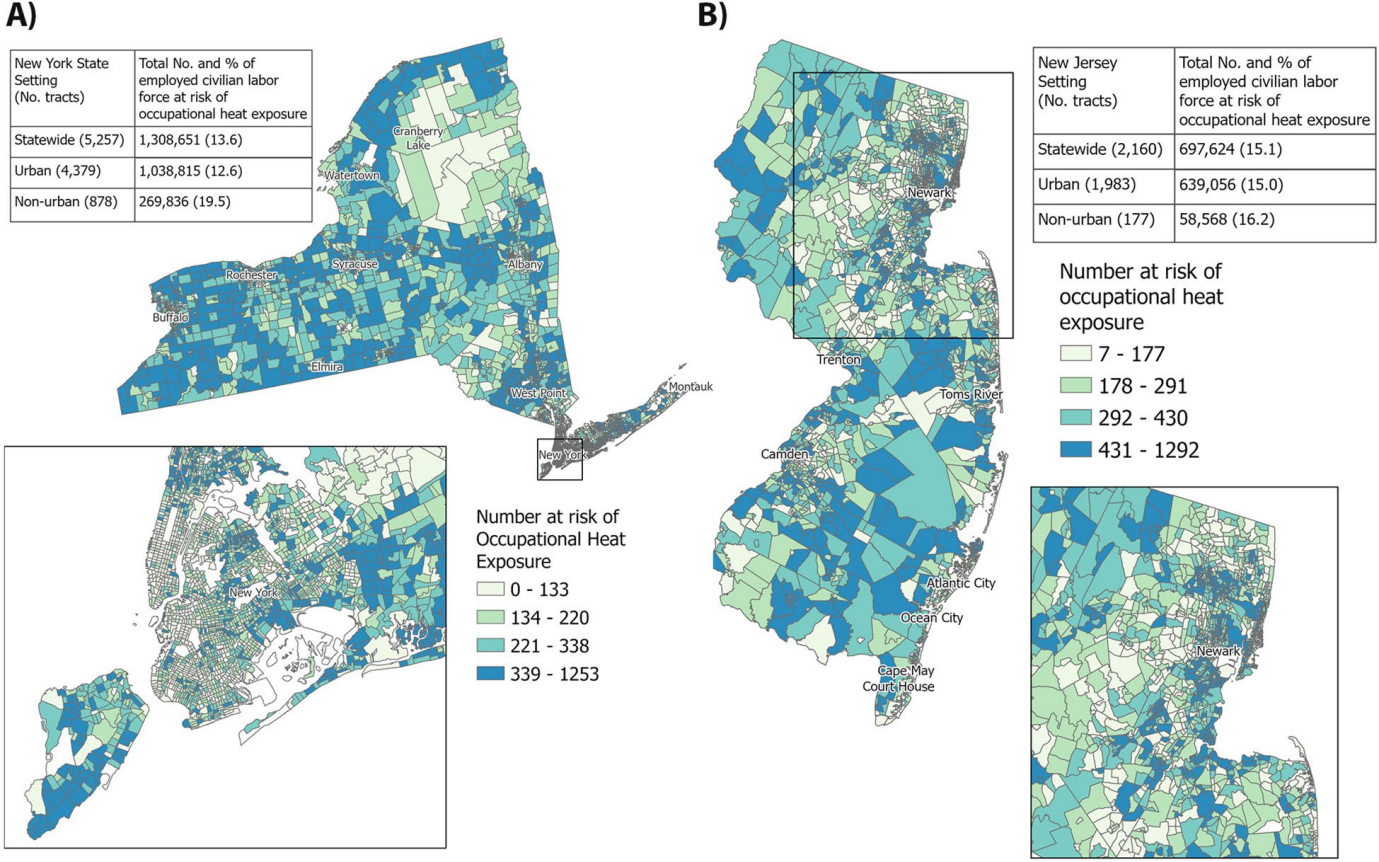
Number and geographic location of workers at risk of occupational heat exposure by census tract in **A**) New York and **B**) New Jersey. The maps display the population density of employed residents at risk of heat exposure (outdoors or indoors) for at least one day per week as part of their job. See methods for data sources. Maps were created in ArcGIS Pro (version 3.5.2)

Of the total civilian employed populations in NYS (9.6 million) and NJ (4.6 million), 13.6% and 15.1% respectively were found to have an elevated risk of occupational heat exposure (Fig. 1). In NYS, a higher proportion of workers residing in non-urban areas (19.5%) compared to urban areas (12.6%) had elevated heat risk (Fig. 1(A)) although localized hotspots existed in Staten Island, the Bronx, Queens, and Brooklyn. Nearly the entire population in NJ was classified as urban (Fig. 1(B)).

### Intersection of occupational heat exposure with social, health, and environmental factors

#### Census-tract distribution

Both NYS and NJ had higher proportions of outdoor heat exposed employed residents (NYS: 12.2%, NJ: 12.6%) compared to those exposed to indoor heat (NYS: 4.1%, NJ:4.9%; Tables 3 and 4). The most prevalent social determinants were people of color (NYS: 45.4%, NJ: 47.2%) and low income populations (NYS: 28.8%, NJ: 22.6%) (Tables 3 and 4). Urban tracts in both states had higher average percentages of people of color, low-income populations, individuals with less than a high school education, and those with limited English proficiency, while non-urban tracts had a higher proportion of adults aged 65 and older. Health indicators showed modest urban–non-urban differences in both states, with non-urban tracts in NYS having slightly higher average prevalence of CHD (rural-7.8%, urban-6.2%), COPD (rural-7.8%, urban-5.9%), and high blood pressure (rural-34.8%, urban-30.8%). Air quality indicators also varied by urbanicity, with urban areas showing higher average levels of ozone and NO₂. The average annual frequency of heat waves was low overall (NYS: 0.9, NJ:2.4), particularly in NYS non-urban tracts (0.3).

**Table 3:** Distribution of occupational, social, health, and environmental heat vulnerability domain factors by census tract in New York stratified by urbanicity

Vulnerability Domain	Total: New York State (*N* = 5,257)		Urban (≥ 75% urban population; *N* = 4,379 Tracts)		Non-Urban (< 75% urban population; *N* = 878 Tracts)	
**Characteristic**	**Mean (SD)**	**Median (min, max)**	**Mean (SD)**	**Median (min, max)**	**Mean (SD)**	**Median (min, max)**
Occupational						
Outdoor Heat Exposure Percent	12.2 (5.5)	12.1 (0, 40.3)	11.3 (5.3)	11.2 (0, 40.3)	16.6 (4.6)	16.8 (1.7, 33.8)
Indoor Heat Exposure Percent	4.1 (2.4)	3.8 (0, 21)	3.6 (2)	3.4 (0, 21)	6.7 (2.2)	6.7 (0.9, 14.3)
Heat Exposure (outdoor or indoor) Percent	14.3 (6.4)	14.1 (0, 47)	13.1 (5.9)	13.2 (0, 47)	20 (5.5)	20.4 (4.6, 37.3)
Social						
People of Color Percent	45.4 (33.2)	36.8 (0, 100)	52.2 (31.9)	47.1 (0, 100)	11.3 (11.2)	7.9 (0.2, 100)
Individuals Over Age 64 Percent	17.4 (7.4)	16.9 (0, 91)	16.6 (7.4)	15.9 (0, 91)	21.2 (6.1)	20.5 (0, 49.2)
Low Income Percent	28.8 (17.7)	25.7 (0, 100)	29.6 (18.7)	26 (0, 100)	25 (10.5)	24.7 (0, 66.3)
Less than High School Education Percent	12.6 (9.9)	9.8 (0, 66.7)	13.5 (10.4)	10.7 (0, 66.7)	8.2 (4.8)	7.3 (0, 36.4)
Limited English-Speaking Percent	7.7 (10.9)	3.1 (0, 74.9)	9.1 (11.4)	4.5 (0, 74.9)	0.8 (1.5)	0 (0, 14.3)
Health (crude prevalence among adults)						
Coronary Heart Disease	6.5 (1.7)	6.3 (0.4, 24.7)	6.2 (1.6)	6.1 (0.4, 24.7)	7.8 (1.3)	7.8 (0.9, 14.3)
Chronic Obstructive Pulmonary Disease	6.2 (2.2)	5.9 (0.8, 19.7)	5.9 (2.1)	5.6 (0.8, 19.7)	7.8 (1.7)	7.8 (1.9, 14.3)
Current Asthma	10.5 (1.5)	10.5 (6.3, 18.4)	10.4 (1.6)	10.3 (6.3, 18.4)	10.9 (0.7)	10.9 (8.1, 14.5)
Diabetes	11.2 (3.1)	10.9 (0.7, 28.6)	11.2 (3.4)	10.8 (0.7, 28.6)	11.0 (1.6)	11 (1.5, 19.8)
High Blood Pressure	31.5 (5.9)	31.8 (4.1, 56.6)	30.8 (6.1)	30.9 (4.1, 56.6)	34.8 (3.6)	35 (7.7, 45)
Environment						
Particulate Matter 2.5 (μg m^3^)	7.4 (0.6)	7.6 (5, 8.4)	7.6 (0.5)	7.7 (5, 8.4)	6.6 (0.6)	6.6 (5, 8)
Ozone (PPM)	62 (5.6)	64.6 (50.7, 72.7)	63.4 (4.7)	65.1 (50.8, 72.7)	54.9 (3.8)	53.7 (50.7, 71.8)
Nitrogen Dioxide (PPB)	10.2 (5.1)	9.6 (1.4, 25.3)	11.4 (4.7)	11.1 (1.7, 25.3)	4.1 (1)	4.1 (1.4, 9.1)
Heat Wave—Annualized Frequency	0.9 (0.6)	0.9 (0, 7.4)	1.1 (0.6)	1.1 (0, 7.4)	0.3 (0.2)	0.3 (0, 0.9)

**Table 4:** Distribution of occupational, social, health, and environmental heat vulnerability domain factors by census tract in New Jersey stratified by urbanicity

Vulnerability Domain	Total: New Jersey (*N* = 2,160 Tracts)		Urban (≥ 75% urban population; *N* = 1,983 Tracts)		Non-Urban (< 75% urban population; *N* = 177 Tracts)	
**Characteristic**	**Mean (SD)**	**Median**	**Mean (SD)**	**Median**	**Mean (SD)**	**Median**
Occupational						
Outdoor Heat Exposure Percent	12.6 (6.1)	12.2 (0.6, 51.5)	12.5 (6.2)	12 (0.6, 51.5)	14.1 (5.4)	14 (1.5, 35.1)
Indoor Heat Exposure Percent	4.9 (2.6)	4.6 (0.1, 19.4)	4.8 (2.6)	4.6 (0.1, 17.5)	5.7 (2.6)	5.6 (0.8, 19.4)
Heat Exposure (outdoor or indoor) Percent	15.6 (7.7)	15 (1.1, 56.9)	15.5 (7.8)	14.8 (1.1, 56.9)	16.8 (6.2)	16.8 (3, 36)
Social						
People of Color Percent	47.2 (29.5)	41.5 (0, 100)	49.6 (29.3)	45.3 (0, 100)	19.9 (12.5)	16.7 (0.9, 76.8)
Individuals Over Age 64 Percent	17.1 (9.3)	15.7 (0, 82.5)	17 (9.5)	15.4 (0, 82.5)	18.6 (6.5)	18.2 (0.4, 55.1)
Low Income Percent	22.6 (16.8)	17.4 (0, 88.7)	23.3 (17.1)	18.2 (0, 88.7)	14.4 (8.7)	12.8 (1.5, 52.1)
Less than High School Education Percent	9.8 (9.2)	6.9 (0, 58)	10.2 (9.4)	7.1 (0, 58)	6 (4.5)	4.9 (0, 30.6)
Limited English-Speaking Percent	7.1 (9.1)	3.6 (0, 62.7)	7.6 (9.3)	4 (0, 62.7)	1.3 (1.9)	0.7 (0, 14)
Health (crude prevalence among adults)						
Coronary Heart Disease	6.2 (1.7)	6 (0.7, 17.2)	6.1 (1.7)	5.9 (0.9, 17.2)	6.7 (1.3)	6.7 (0.7, 12.5)
Chronic Obstructive Pulmonary Disease	5.5 (1.8)	5.3 (0.8, 15.6)	5.5 (1.8)	5.3 (0.8, 15.6)	6.0 (1.5)	5.9 (1.5, 11.8)
Current Asthma	9.6 (1.3)	9.4 (5.7, 15)	9.5 (1.4)	9.4 (5.7, 15)	9.7 (0.8)	9.7 (7.4, 11.8)
Diabetes	10.5 (2.8)	10 (1.2, 26.4)	10.6 (2.9)	10.1 (2.1, 26.4)	9.6 (1.6)	9.4 (1.2, 17.2)
High Blood Pressure	31 (5.8)	30.5 (7.7, 57)	30.9 (5.9)	30.3 (9.2, 57)	32.2 (3.9)	32.1 (7.7, 45.6)
Environment						
Particulate Matter 2.5 (μg m^3^)	7.6 (0.5)	7.6 (6.5, 8.4)	7.7 (0.5)	7.7 (6.5, 8.4)	7.3 (0.3)	7.2 (6.6, 8)
Ozone (PPM)	61.4 (2.1)	61.6 (53.9, 65.7)	61.6 (1.9)	61.8 (55.1, 65.7)	58.6 (2.2)	58.6 (53.9, 64.2)
Nitrogen Dioxide (PPB)	8.9 (4)	7.9 (2.1, 19.3)	9.3 (4)	8.5 (2.3, 19.3)	4.6 (1)	4.7 (2.1, 7.1)
Heat Wave—Annualized Frequency	2.4 (1.9)	1.7 (0.3, 9.2)	2.4 (1.9)	1.7 (0.3, 9.2)	2.1 (2.1)	1.2 (0.6, 6.4)

#### Correlations

**Fig. 2:**
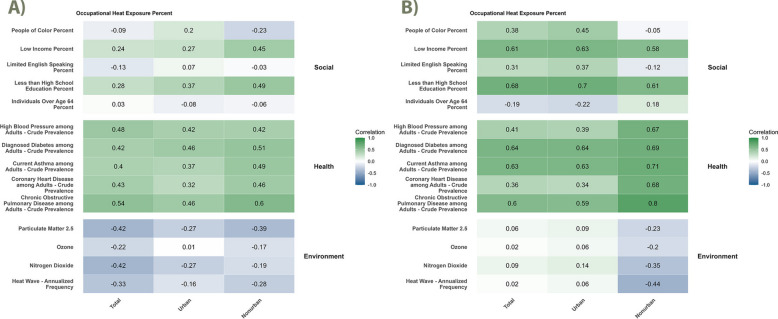
Correlations between occupational heat exposure and social, health, and environmental vulnerability factors in **A**) New York and **B**) New Jersey. Heat maps illustrate Spearman correlations between occupational heat exposure and selected vulnerability factors, with results stratified by urbanicity. See methods for data sources. Maps were created in ArcGIS Pro (version 3.5.2)

Occupational heat exposure risk among employed residents was correlated with social and health vulnerabilities in both NYS and NJ, but tended to have weaker or negative correlations with environmental exposures (Fig. 2; see Supplemental Tables 3 and 4 for outdoor and indoor correlations shown separately in NY and NJ, respectively). In some cases, statewide correlation coefficients fell outside the range of the corresponding urban and non-urban estimates, suggesting potential confounding by urbanicity.

Occupational heat exposure showed a mixed relationship with social determinants in NYS, especially in non-urban areas (Fig. 2(A)). The strongest correlations were observed for the percentage of residents with less than a high school education (ρ = 0.37 urban, ρ = 0.49 non-urban) and low income (ρ = 0.27 urban, ρ = 0.45 non-urban). The percentage of people of color showed a positive correlation in urban areas but a negative one in non-urban areas. In contrast, percent over age 64 and limited English proficiency showed weak or no associations.

In NJ, occupational heat exposure was strongly correlated with multiple social determinants in both urban and non-urban areas (Fig. 2(B)). The strongest correlations were observed for percent with less than a high school education (ρ = 0.70 urban, ρ = 0.61 non-urban) and low income (ρ = 0.63 urban, ρ = 0.58 non-urban). Limited English proficiency and percent people of color also showed moderate positive correlations in urban areas (ρ = 0.37 and ρ = 0.45, respectively), but were weakly or negatively correlated in non-urban areas. Percent over age 64 showed weak or inconsistent correlations among urban and non-urban areas.

All health indicators were positively correlated with occupational heat exposure in both states (Fig. 2). IN NYS, correlations were modest to strong, particularly for COPD (ρ = 0.46 urban, ρ = 0.60 non-urban), diabetes (ρ = 0.46 urban, ρ = 0.51 non-urban), and CHD (ρ = 0.32 urban, ρ = 0.46 non-urban) (Fig. 2(A)). In NJ, health indicators showed even stronger positive correlations, with particularly high coefficients for COPD (ρ = 0.80 non-urban, ρ = 0.59 urban), asthma (ρ = 0.71 non-urban, ρ = 0.63 urban), and diabetes (ρ = 0.69 non-urban, ρ = 0.64 urban) (Fig. 2(B)).

Environmental exposures were generally negatively or weakly correlated with occupational heat exposure in both states (Fig. 2). In NYS, the strongest negative correlations were observed for PM_2.5_ and NO_2_ (both ρ = –0.42 in total areas). In NJ, environmental exposures were weakly or negatively correlated overall, particularly in non-urban areas.

#### Geographic overlap

**Fig. 3:**
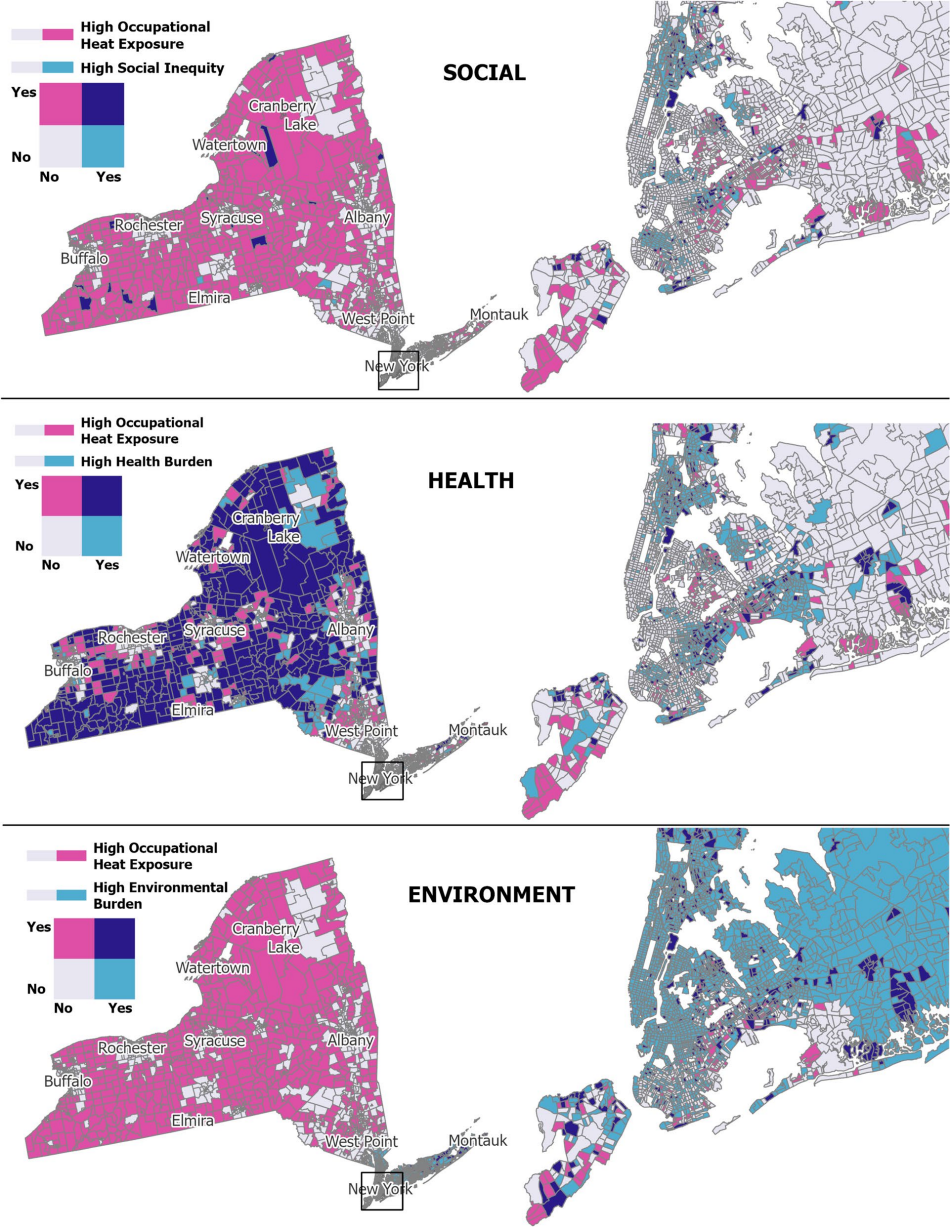
Spatial Overlap of Census Tracts in the Highest Quintile of Occupational, Social, Health, and Environmental Vulnerability, New York State. Map show NYS census tracts in the top quintile for four vulnerability domains: occupational heat (workers exposed to indoor or outdoor heat ≥ 1 day/week), social (demographic and socioeconomic factors), health (chronic disease prevalence), and environmental (air pollution and heat wave frequency). See Methods for detailed definitions and data sources. Maps created in ArcGIS Pro 3.5.2

**Fig. 4:**
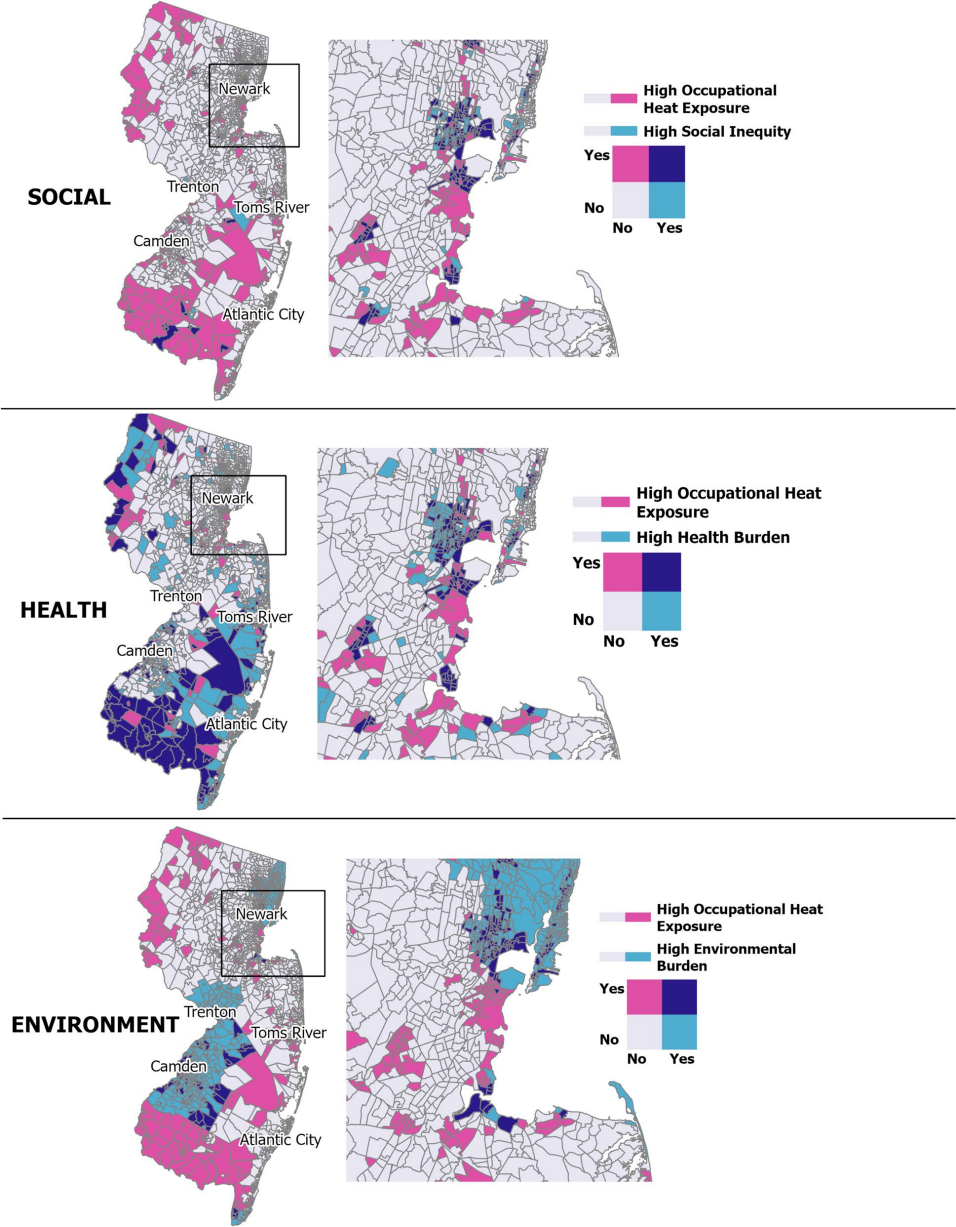
Spatial Overlap of Census Tracts in the Highest Quintile of Occupational, Social, Health, and Environmental Vulnerability, New Jersey. Map shows NJ census tracts in the top quintile for the same vulnerability domains described in Fig. 3. See Methods for detailed definitions and data sources. Maps created in ArcGIS Pro 3.5.2

Overlap between occupational heat and social vulnerability was modest overall (Figs. 3 and 4). In NYS, 15.9% (*n* = 252) of occupationally heat vulnerable tracts (*n* = 1,584) also met criteria for social vulnerability (Table 5, Fig. 3). Using less restrictive social vulnerability criteria (i.e., ranking ≥ 80th percentile for low-income alone), would have increased the occupational-social vulnerability overlap by only 6 percentage points, with similar proportional increases across urbanicity (data not shown). In NJ, this overlap was higher at 44.1% (272 of 617), concentrated largely in the densely populated area around Newark, a major New York City (NYC) commuter hub (Table 5, Fig. 4).

**Table 5:** Overlap in occupational, social, health, and environmental factors contributing to heat vulnerability by census tracts in New York and New Jersey

	**NEW YORK STATE**		**NEW JERSEY**	
	**Total (*n* = 5,257)**	**Occupational Heat Vulnerability**^**a**^	**Total (*n* = 2160)**	**Occupational Heat Vulnerability**
**Domains**	**No of Tracts (%)**	**No of Tracts (%)**	**No of Tracts (%)**	**No of Tracts (%)**
Social vulnerability^b^	856 (16.3)	252 (15.9)	398 (18.4)	272 (44.1)
Health vulnerability^c^	2254 (42.9)	985 (62.2)	864 (40.0)	429 (69.5)
Environment vulnerability^d^	2628 (50.0)	468 (29.5)	986 (45.6)	323 (52.4)
Total Tracts	5257 (100.0)	1584	2160 (100.0)	617

^a^A tract is considered to have an occupational heat vulnerability if it is at or above the 80th percentile for the proportion of workers exposed to indoor or outdoor heat

^b^A tract is considered to have a social vulnerability if it is at or above the 80th percentile for percentage of people of color, population aged 65 or older, less than high school education, or limited English proficiency *and* low-income percent

^c^A tract is considered to have a health vulnerability if it is at or above the 80th percentile for coronary heart disease, chronic obstructive pulmonary disease, current asthma, diabetes, or high blood pressure among adults

^d^A tract is considered to have an environmental vulnerability if it is at or above the 80th percentile for particulate matter 2.5, ozone, nitrogen dioxide, or annualized heat wave frequency

Convergence between occupational heat and health vulnerabilities was stronger in both states. In NYS, 62.2% of occupationally heat vulnerable tracts were also classified as demonstrating health vulnerability (Table 5), with clusters primarily in Upstate NY, Long Island, and the outer boroughs of NYC (Fig. 3). In NJ, 69.5% of occupationally heat vulnerable tracts also showed health vulnerability (Table 5), with overlaps spanning urban centers as well as the less populated northeastern and southern borders (Fig. 4).

Environmental vulnerability showed limited overlap with occupational heat risk in NYS but greater alignment in NJ. In NYS, 17.8% (468 of 2,628) of environmentally vulnerable tracts were also occupationally heat vulnerable (Table 5), with environmentally vulnerable tracts concentrated in the southern portion of the state, particularly NYC and its surrounding counties (Fig. 3). In NJ, the alignment was more pronounced, with 52.4% of environmentally vulnerable tracts (*n* = 986) facing elevated occupational heat risk, especially around Newark (which also showed overlap with social and health vulnerabilities), Trenton, and Camden (Philadelphia commuter city) (Table 5). Findings were robust to alternative percentile thresholds, with similar spatial patterns observed at the ≥ 70th and ≥ 90th percentiles.

## Discussion

This study developed and applied a replicable method for generating census tract-level estimates of occupational heat exposure risk for employed residents of NYS and NJ using publicly available data. We demonstrated the significance of these estimates by analyzing their relationship with social, health, and environmental indicators associated with heat-related morbidity and mortality and routinely used in existing heat vulnerability indices. Approximately 1.3 million employed residents in NYS (13.6% of total employed) and 698,000 in NJ (15.1% of total employed) were employed in occupations with elevated occupational heat exposure risk, with higher proportions in less urbanized areas. At the census tract level, occupational heat exposure risk among employed residents was most strongly correlated with health vulnerability, followed by social vulnerability; environmental indicators showed weak or negative correlations. Geographic overlap of tracts in the highest quintiles of occupational risk and other vulnerability domains varied but was modest overall; occupational and health vulnerabilities aligned in less urban areas, while occupational and social and environmental vulnerabilities overlapped near urban centers. These distinct spatial patterns emphasize the value of incorporating occupation as a separate dimension of risk in heat vulnerability mapping to guide future research directions and support targeted geographically-based interventions in community and workplace settings, especially in the absence of enforceable labor protections against extreme heat.

The moderate correlation strength and partial geographic alignment between occupational and other vulnerabilities suggest that current vulnerability mapping approaches do not silently address the occupational heat exposure threat. This omission is particularly concerning given the uncertainty around establishing a federal heat standard and the patchwork of state rules, [75] leaving nearly 2 million high-risk workers in NYS and NJ and an estimated 36 million workers nationwide without enforceable protections. [27] While health departments provide limited surveillance and guidance on occupational heat exposure, [76, 77] these efforts lack enforcement mechanisms and can be disconnected from broader public health messaging (e.g., “stay indoors”).

As state and local government agencies work to mitigate climate change vulnerability, [26, 78, 79] our study results demonstrate the feasibility and importance of additional attention to occupational heat exposure. The availability of high-resolution data on the number, location, and occupational composition of heat exposed residents can support prevention efforts that operate at both community and workplace settings. These tract-level estimates provide a policy-relevant evidence base to support worker protections, inform heat standards, guide outreach, and prioritize resource allocation in communities where occupational exposure is concentrated.

The observed discordance between occupational heat and social vulnerability, especially in NYS and outside of urban areas, challenges the sole use of income and education as proxies for occupational risk in epidemiologic research and mapping tools. Workers across the wage spectrum, including low- (e.g., farmworkers, ambulance attendants), middle- (e.g., electricians and postal mail carriers, patrol officers), and high-wage (e.g. farm and construction managers) occupations, demonstrated elevated heat exposure risk. Because heat and other widespread environmental hazards affect diverse occupations, traditional assumptions that occupational vulnerability is concentrated among low-wage or less-skilled jobs, in the context of climate change warrant reconsideration.

Interestingly, we observed strong correlations and a notable geographic overlap between tracts with high occupational heat exposure and high prevalence of chronic health conditions but with low to moderate social vulnerability, particularly in less urban areas. While our study cannot establish causality or confirm population overlap, prior research has demonstrated an increased risk of hospitalizations due to heat-related illness and mortality among rural and suburban clusters in comparison to urbanized areas. [24, 80] This pattern is also consistent with evidence demonstrating a positive association between blue collar occupations, prevalent in rural areas, and rates of smoking, cardiovascular diseases, and all-cause mortality. [81–84] Occupational heat exposure may exacerbate existing chronic health conditions, increasing the risk of heat-related fatalities. [85] These findings highlight the added value of implementing workplace heat protections for all workers, especially in regions like upstate NY, where occupational heat exposure and chronic health conditions are both prevalent. In such areas, increased awareness on heat risk and potential interactions with medications that disrupt thermoregulation of fluid-balance is warranted.

In socially vulnerable communities already prioritized for heat mitigation, more detailed occupational data can enhance existing efforts. The greatest overlap between social and occupational heat vulnerability occurred in densely populated urban areas of NY and NJ; a pattern that may have been driven by residential segregation and discriminatory hiring practices. [86, 87] Many employed residents in low-wage, heat exposed jobs (e.g., delivery workers, day laborers, farmworkers, warehouse packers and sorters) lack union protections, structured breaks, and fear retaliation for refusing unsafe work. [88] These communities may therefore benefit from targeted outreach (e.g., “know your rights” trainings and distribution of water and cooling gear), streamlined eligibility for air conditioning assistance programs, and policies such as paid sick leave during extreme heat events, as implemented in Oregon. [89, 90].

The weak or negative correlations between occupational heat exposure and environmental vulnerability likely indicate divergent geographic patterns. While heat waves and air pollution were clustered in urban areas, occupational heat exposure was more prevalent in less-urban areas. These observed differences reinforce the utility of distinct vulnerability assessments for urban and rural regions. However, the urban–rural environmental risk differences may diminish over time as wildfires and extreme heat expand across regions previously considered less at risk. [91, 92].

This study develops tract-level estimates capturing the occupational heat exposure of employed residents, producing the first geographically detailed dataset of its kind for NYS and NJ. This dataset expands on prior studies that relied on regional BLS data [38, 93] by providing finer geographic granularity and including workers in nonfarm industries, the self-employed, and undocumented populations. By leveraging publicly available data, this approach is readily adaptable to other states seeking to examine occupational heat vulnerability alongside other tract-level indicators reflecting disproportionate burden. Although recent changes to several widely used federal composite indices limit direct comparisons with federal tools, this framework remains well suited to exploratory analyses of how occupational heat exposure co-occurs with these indicators. [94, 95] These tract-level estimates are a timely resource for evidence-based action amid rising temperatures, limited workplace protections, and growing efforts to integrate occupational risk into public health surveillance, accelerated by the COVID-19 pandemic. [42].

This study has several limitations. Our occupational exposure metric relied on reported frequency of heat exposure, without accounting for radiant heat or modifying factors like personal protective equipment (PPE) use, physical exertion, or break access. Nonetheless, our high-risk occupation list closely matched one developed using a more refined methodology, differing in only one borderline case (i.e., Floor, ceiling and wall insulation workers). [93] Exposure misclassification may have also occurred by assigning PUMA-level occupational distributions to nested tracts, especially in dense urban areas with sharp socioeconomic contrasts. Using tract-level major occupation group estimates, the only approach we have seen for incorporating occupation into a limited number of climate and social vulnerability indices, [96–98] would have introduced even greater misclassification due to wide variation in heat exposure within those broad categories. Importantly, PUMAs align closely with NYC community districts, which are designed to capture areas with similar built and socioeconomic characteristics. [99].

Our binary urbanicity classification could not account for intra-urban variability in built- and natural-environmental factors like surface temperature, which are best identified at the block group level. [54, 56] We further limited our analysis to a focused group of social, health, and environmental indicators and excluded resilience factors entirely, including access to air conditioning, a strong predictor of heat-related morbidity and mortality, [59] due to inconsistent tract-level data across urban and non-urban settings. Additionally, our definition of a “vulnerable” tract using the upper-quintile threshold was inherently arbitrary; examining alternative thresholds and the full continuum of risk might provide a better understanding of vulnerability. Finally, as an ecological study, our analysis cannot establish causality or link occupational heat exposure to individual-level health outcomes.

Future research can build on these and similar methods [93] to generate tract-level estimates of occupational exposure to heat and other climate-related hazards across U.S. states and territories using ACS microdata. Because the framework relies on publicly available data sources, standardized occupational classifications, and clearly described methods, it can be readily replicated and applied in other geographic settings. It is also adaptable to region-specific and co-occurring risks such as wildfire smoke or flooding.

Additional priorities include evaluating how incorporating occupational factors affects existing heat vulnerability indices and conducting ecological and observational studies to assess associations with heat-related emergency department visits, hospitalizations, and fatalities, particularly under joint exposure to heat and air pollution.

## Conclusions

Integrating occupation into climate and social vulnerability indices is important, because it is an essential human activity, a social determinant of health, and a salient source of heat exposure that is not fully captured by existing social, health, and environmental data. Our findings demonstrate the distinct yet intersecting relationship between occupational heat exposure and other vulnerability domains, laying the groundwork for future research, providing a method usable in other jurisdictions, and offering insights to inform heat prevention strategies in both community and workplace settings.

## Supplementary Information

Supplementary Material 1.

Supplementary Material 2.

## Data Availability

A dataset supporting the conclusions of this article is included within the article’s additional files.

## Abbreviations

ACS: American Community Survey
BLS: Bureau of Labor Statistics
CDC: Centers for Disease Control and Prevention
CHD: Coronary Heart Disease
COPD: Chronic Obstructive Pulmonary Disease
EPA: Environmental Protection Agency
FEMA: Federal Emergency Management Agency
HBP: High Blood Pressure
NJ: New Jersey
NYS: New York State
O*NET: Occupational Information Network
OSHA: Occupational Safety and Health Administration
PUMA: Public Use Microdata Area
PUMS: Public Use Microdata Sample
SOC: Standard Occupational Classification

## References

[1] Hayden M, Schramm P, Beard C, Bell J, Bernstein A, Bieniek-Tobasco A, et al. Human health. In Fifth National Climate Assessment. U.S. Global Change Research Program. 2023. https://nca2023.globalchange.gov/chapter/15/.

[2] Intergovernmental Panel on Climate Change (IPCC). Climate Change 2022: Impacts, Adaptation, and Vulnerability. Contribution of Working Group II to the Sixth Assessment Report of the Intergovernmental Panel on Climate Change. 2022. Available online at: https://www.ipcc.ch/report/sixth-assessment-report-working-group-ii/.

[3] Meehl GA, Tebaldi C. More intense, more frequent, and longer lasting heat waves in the 21st century. Science. 2004;305(5686):994–7. 15310900 10.1126/science.1098704

[4] Solar O, Irwin A. A conceptual framework for action on the social determinants of health. Social Determinants of Health Discussion Paper 2 (Policy and Practice). Geneva: World Health Organization; 2010. ISBN: 978-92-4-150085-2. https://www.who.int/publications/i/item/9789241500852.

[5] Hacker K, Auerbach J, Ikeda R, Philip C, Houry D. Social determinants of health-an approach taken at CDC. J Public Health Manag Pract. 2022;28(6):589–94. 36194813 10.1097/PHH.0000000000001626 PMC9555578

[6] Flynn MA, Check P, Steege AL, Sivén JM, Syron LN. Health Equity and a Paradigm Shift in Occupational Safety and Health. Int J Environ Res Public Health. 2021;19(1):349. 10.3390/ijerph19010349. 10.3390/ijerph19010349 PMC8744812 35010608

[7] Applebaum KM, Graham J, Gray GM, LaPuma P, McCormick SA, Northcross A, et al. An overview of occupational risks from climate change. Curr Environ Health Rep. 2016;3(1):13–22. 26842343 10.1007/s40572-016-0081-4

[8] Schulte PA, Jacklitsch BL, Bhattacharya A, Chun H, Edwards N, Elliott KC, et al. Updated assessment of occupational safety and health hazards of climate change. J Occup Environ Hyg. 2023;20(5-6):183–206. 10.1080/15459624.2023.2205468. 10.1080/15459624.2023.2205468 PMC10443088 37104117

[9] Zuzak C, Mowrer M, Goodenough E, Burns J, Ranalli N, Rozelle J. The national risk index: establishing a nationwide baseline for natural hazard risk in the US. Nat Hazards. 2022;114(2):2331–55.

[10] Federal Emergency Management Agency (FEMA). The National Risk Index 2023 [Available from: https://hazards.fema.gov/nri/.

[11] Flanagan BE, Gregory EW, Hallisey EJ, Heitgerd JL, Lewis B. A social vulnerability index for disaster management. J Homeland Sec Emerg Manage. 2011;8(1). 10.2202/1547-7355.1792.

[12] Agency for Toxic Substances and Disease Registry (ATSDR). CDC/ATSDR Social Vulnerability Index 2024 [Available from: https://www.atsdr.cdc.gov/placeandhealth/svi/index.html.

[13] U.S. Environmental Protection Agency (EPA). Environmental Justice Screening and Mapping Tool V.2.2 (EJScreen) 2022 [Available from: https://www.epa.gov/ejscreen; https://19january2021snapshot.epa.gov/ejscreen_.html.

[14] White House Council on Environmental Quality. Climate and Economic Justice Screening Tool (CEJST) 2022 [Available from: https://screeningtool.geoplatform.gov/en/#3/33.47/-97.5.

[15] Manware M, Dubrow R, Carrión D, Ma Y, Chen K. Residential and race/ethnicity disparities in heat vulnerability in the United States. Geohealth. 2022;6(12):e2022GH000695. 10.1029/2022GH000695 PMC9744626 36518814

[16] Nayak SG, Shrestha S, Kinney PL, Ross Z, Sheridan SC, Pantea CI, et al. Development of a heat vulnerability index for New York State. Public Health. 2018;161:127–37. 29195682 10.1016/j.puhe.2017.09.006

[17] New York City Department of Health and Mental Hygiene. Interactive Heat Vulnerability Index 2020 [Available from: https://a816-dohbesp.nyc.gov/IndicatorPublic/data-features/hvi/.

[18] U.S. Global Change Research Program. The Impacts of Climate Change on Human Health in the United States: A Scientific Assessment. Washington, DC: U.S. Global Change Research Program; 2016. p. 312.

[19] Balbus JM, Malina C. Identifying vulnerable subpopulations for climate change health effects in the United States. J Occup Environ Med. 2009;51(1):33–7. 19136871 10.1097/JOM.0b013e318193e12e

[20] Gamble JL, Schmeltz MT, Hurley B, Hsieh J, Jette G, Wagner H. Mapping the Vulnerability of Human Health to Extreme Heat in the United States. Available online at: https://assessments.epa.gov/risk/document/&deid%3D341861: U.S. Environmental Protection Agency,; 2018.

[21] U.S. Environmental Protection Agency (EPA). Climate Change and Social Vulnerability in the United States: A Focus on Six Impacts. U.S. Environmental Protection Agency, EPA 430-R-21–003. 2021. Available online at: https://www.epa.gov/cira/social-vulnerability-report.

[22] Gronlund CJ. Racial and socioeconomic disparities in heat-related health effects and their mechanisms: a review. Curr Epidemiol Rep. 2014;1(3):165–73. 25512891 10.1007/s40471-014-0014-4 PMC4264980

[23] Reid CE, O’Neill MS, Gronlund CJ, Brines SJ, Brown DG, Diez-Roux AV, et al. Mapping community determinants of heat vulnerability. Environ Health Perspect. 2009;117(11):1730–6. 20049125 10.1289/ehp.0900683 PMC2801183

[24] Schmeltz MT, Sembajwe G, Marcotullio PJ, Grassman JA, Himmelstein DU, Woolhandler S. Identifying individual risk factors and documenting the pattern of heat-related illness through analyses of hospitalization and patterns of household cooling. PLoS ONE. 2015;10(3):e0118958. 25742021 10.1371/journal.pone.0118958 PMC4351173

[25] O’Neill MS, Zanobetti A, Schwartz J. Modifiers of the temperature and mortality association in seven US cities. Am J Epidemiol. 2003;157(12):1074–82. 12796043 10.1093/aje/kwg096

[26] Spector JT, Masuda YJ, Wolff NH, Calkins M, Seixas N. Heat exposure and occupational injuries: review of the literature and implications. Curr Environ Health Rep. 2019;6(4):286–96. 31520291 10.1007/s40572-019-00250-8 PMC6923532

[27] Occupational Safety and Health Administration. Heat injury and illness prevention in outdoor and indoor work settings (Proposed Rule). Available online at: https://www.federalregister.gov/d/2024-14824: Federal Register; 2024.

[28] Gubernot DM, Anderson GB, Hunting KL. Characterizing occupational heat-related mortality in the United States, 2000–2010: an analysis using the census of fatal occupational injuries database. Am J Ind Med. 2015;58(2):203–11. 25603942 10.1002/ajim.22381 PMC4657558

[29] Dong XS, West GH, Holloway-Beth A, Wang X, Sokas RK. Heat-related deaths among construction workers in the United States. Am J Ind Med. 2019;62(12):1047–57. 31328819 10.1002/ajim.23024

[30] Calkins MM, Bonauto D, Hajat A, Lieblich M, Seixas N, Sheppard L, et al. A case-crossover study of heat exposure and injury risk among outdoor construction workers in Washington State. Scand J Work Environ Health. 2019;45(6):588–99. 30869152 10.5271/sjweh.3814

[31] Amoadu M, Ansah EW, Sarfo JO, Hormenu T. Impact of climate change and heat stress on workers’ health and productivity: a scoping review. J Climate Change Health. 2023:100249. 10.1016/j.joclim.2023.100249.

[32] Mullins-Jaime C. Trending occupational fatalities and injuries: an assessment of projected climate change related impacts in the United States since 1992. Int J Environ Res Public Health. 2023;20(13):6258. 37444106 10.3390/ijerph20136258 PMC10341741

[33] Hawkins D, Ibrahim M. Characteristics of occupational environmental heat injuries/illnesses, Survey of Occupational Injuries and Illnesses, 2011 to 2019. J Occup Environ Med. 2023;65(3):e146–e152. 10.1097/JOM.0000000000002794. 10.1097/JOM.0000000000002794 36727985

[34] Jay A, Reidmiller D, Avery C, Barrie D, DeAngelo B, Dave A, et al. The Fourth National Climate Assessment: Summary Findings and Overview. ESS Open Archive. 2019. 10.1002/essoar.10500761.1.

[35] Dahl K, Licker R. Too Hot to Work. Cambridge, MA: Union of Concerned Scientists; 2021. 10.47923/2021.14236.

[36] New Jersey Department of Health. Climate Change: Protection of Outdoor Workers 2025 [Available from: https://www.nj.gov/health/ceohs/public-health-tracking/climate-change/.

[37] New York State Department of Labor (NYS DOL). Extreme Weather Guidance to Protect Outdoor Workers Available online at: https://dol.ny.gov/extreme-weather-guidance2024 [Available from: https://dol.ny.gov/extreme-weather-guidance.

[38] Office of the New York City Comptroller. Safeguarding Outdoor Workers in a Changing Climate. Available online at: https://comptroller.nyc.gov/reports/safeguarding-outdoor-workers-in-a-changing-climate; 2024.

[39] Davenport C. Extreme heat is testing Biden’s new workplace rules. New York Times [Internet]. 2024 May 25 [cited 2025 Aug 01]; Climate. Available from: https://www.nytimes.com/2024/05/25/climate/extreme-heat-biden-workplace.html.

[40] Gerstein T. Workers shouldn’t have to risk their lives in a heat wave. New York Times [Internet]. 2023 Jul 12 [cited 2026 Mar 09]; Opinion. Available from: https://www.nytimes.com/2023/07/12/opinion/heat-wave-workers-protection.html.

[41] Knowlton K. Occupational Heat Safety Standards in the United States 2025 [Available from: https://www.nrdc.org/resources/occupational-heat-safety-standards-united-states.

[42] Armenti K, Sweeney MH, Lingwall C, Yang L. Work: a social determinant of health worth capturing. Int J Environ Res Public Health. 2023;20(2). 10.3390/ijerph20021199. 10.3390/ijerph20021199 PMC9859245 36673956

[43] New York State Senate. S.3412: Provides for the regulation of all indoor and outdoor worksites (TEMP Act) Available online at: https://legislation.nysenate.gov/pdf/bills/2025/S34122025 [Available from: https://www.nysenate.gov/legislation/bills/2025/S3412.

[44] New Jersey Legislature. S3884: Establishes "Occupational Heat-Related Illness and Injury Prevention Program" and occupational heat stress standard in DOLWD 2025 [Available from: https://www.njleg.state.nj.us/bill-search/2024/S3884.

[45] U.S. Department of Labor. Occupational Information Network (O*NET version 29.2) 2025 [Available from: https://www.onetonline.org/.

[46] U.S. Bureau of Labor Statistics. O*NET Resource Center: Crosswalk Files 2025 [Available from: https://www.onetcenter.org/crosswalks.html.

[47] U.S. Census Bureau. Table S2401: Occupation by sex for the civilian employed population 16 years and over [Internet]. 2018-2022 American Community Survey 5-Year Estimates. Washington (DC): U.S. Department of Commerce; 2025[cited 2025 Feb 01]. https://data.census.gov/table/ACSST1Y2024.S2401.

[48] Walker K, Herman M. tidycensus: Load Utidycensus: Load US Census Boundary and Attribute Data as 'tidyverse' and 'sf'-Ready Data Frames. R package version 1.7.1. Available online at: https://walker-data.com/tidycensus/2024.

[49] U.S. Census Bureau. 2018-2022 American Community Survey 5-year Public Use Microdata Sample (PUMS) [Internet]. Washington (DC): U.S. Department of Commerce; 2024 Jan 25. https://www.census.gov/programs-surveys/acs/microdata.html.

[50] U.S. Census Bureau. Glossary 2022 [Available from: https://www.census.gov/programs-surveys/geography/about/glossary.html.

[51] U.S. Census Bureau. American Community Survey 2018–2022 5-year PUMS user guide and overview [Internet]. Washington (DC): U.S. Department of Commerce; 2024 Jan[cited 2025 Feb 01]. https://www2.census.gov/programs-surveys/acs/tech_docs/pums/user_guide/pums_user_guide_22.pdf.

[52] Missouri Census Data Center. Geocorr 2022: Geographic correspondence engine [Internet]. Columbia (MO): University of Missouri; 2022 [cited 2025 Feb 01]. https://mcdc.missouri.edu/applications/geocorr2022.html.

[53] Madrigano J, Ito K, Johnson S, Kinney PL, Matte T. A case-only study of vulnerability to heat wave-related mortality in New York City (2000–2011). Environ Health Perspect. 2015;123(7):672–8. 25782056 10.1289/ehp.1408178 PMC4492264

[54] Klein Rosenthal J, Kinney PL, Metzger KB. Intra-urban vulnerability to heat-related mortality in New York City, 1997–2006. Health Place. 2014;30:45–60. 25199872 10.1016/j.healthplace.2014.07.014 PMC4348023

[55] Curriero FC, Heiner KS, Samet JM, Zeger SL, Strug L, Patz JA. Temperature and mortality in 11 cities of the eastern United States. Am J Epidemiol. 2002;155(1):80–7. 11772788 10.1093/aje/155.1.80

[56] Conlon KC, Mallen E, Gronlund CJ, Berrocal VJ, Larsen L, O’Neill MS. Mapping human vulnerability to extreme heat: a critical assessment of heat vulnerability indices created using principal components analysis. Environ Health Perspect. 2020;128(9):97001. 32875815 10.1289/EHP4030 PMC7466325

[57] Cardoza JE, Gronlund CJ, Schott J, Ziegler T, Stone B, O'Neill MS. Heat-related illness is associated with lack of air conditioning and pre-existing health problems in Detroit, Michigan, USA: A Community-Based Participatory Co-Analysis of Survey Data. Int J Environ Res Public Health. 2020;17(16). 10.3390/ijerph17165704. 10.3390/ijerph17165704 PMC7460407 32784593

[58] Schwartz J. Who is sensitive to extremes of temperature?: A case-only analysis. Epidemiology. 2005;16(1):67–72. 15613947 10.1097/01.ede.0000147114.25957.71

[59] O’Neill MS, Zanobetti A, Schwartz J. Disparities by race in heat-related mortality in four US cities: the role of air conditioning prevalence. J Urban Health. 2005;82(2):191–7. 15888640 10.1093/jurban/jti043 PMC3456567

[60] Naughton MP, Henderson A, Mirabelli MC, Kaiser R, Wilhelm JL, Kieszak SM, et al. Heat-related mortality during a 1999 heat wave in Chicago. Am J Prev Med. 2002;22(4):221–7. 11988377 10.1016/s0749-3797(02)00421-x

[61] Centers for Disease Control and Prevention (CDC). Heat-related deaths after an extreme heat event--four states, 2012, and United States, 1999-2009. MMWR Morb Mortal Wkly Rep. 2013;62(22):433–6. 23739336 PMC4604981

[62] Centers for Disease Control and Prevention (CDC). Heat-related deaths--United States, 1999-2003. MMWR Morb Mortal Wkly Rep. 2006;55(29):796–8. 16874294

[63] Centers for Disease Control and Prevention. PLACES: local data for better health, 2022 release [Internet]. Atlanta (GA): U.S. Department of Health and Human Services; 2022. https://www.cdc.gov/places/index.html. [cited 2025 Apr 03].

[64] Hu J, Zeng W, Guo Y, Meng R, Huang S, Zhou C, et al. Modification and mediation effects of ozone on heatwave-mortality association: A time series study in five provinces of China. Environ Pollut. 2025;378:126493. 40398801 10.1016/j.envpol.2025.126493

[65] Analitis A, Michelozzi P, D’Ippoliti D, De’Donato F, Menne B, Matthies F, et al. Effects of heat waves on mortality: effect modification and confounding by air pollutants. Epidemiology. 2014;25(1):15–22. 24162013 10.1097/EDE.0b013e31828ac01b

[66] Ren C, Williams GM, Morawska L, Mengersen K, Tong S. Ozone modifies associations between temperature and cardiovascular mortality: analysis of the NMMAPS data. Occup Environ Med. 2008;65(4):255–60. 17890300 10.1136/oem.2007.033878

[67] Ren C, Williams GM, Mengersen K, Morawska L, Tong S. Temperature enhanced effects of ozone on cardiovascular mortality in 95 large US communities, 1987-2000: assessment using the NMMAPS data. Arch Environ Occup Health. 2009;64(3):177–84. 19864220 10.1080/19338240903240749

[68] Li Y, Ma Z, Zheng C, Shang Y. Ambient temperature enhanced acute cardiovascular-respiratory mortality effects of PM2.5 in Beijing, China. Int J Biometeorol. 2015;59(12):1761–70. 25900003 10.1007/s00484-015-0984-z

[69] Jacob DJ, Winner DA. Effect of climate change on air quality. Atmos Environ. 2009;43(1):51–63.

[70] Kazi DS, Katznelson E, Liu CL, Al-Roub NM, Chaudhary RS, Young DE, et al. Climate change and cardiovascular health: a systematic review. JAMA Cardiol. 2024;9(8):748–57. 38865135 10.1001/jamacardio.2024.1321 PMC11366109

[71] Manson S, Schroeder J, Van Riper D, Knowles K, Terwilliger M, Roberts S. IPUMS National Historical Geographic Information System: 2020 Decennial Census, demographic and housing characteristics [Internet]. Version 18.0. Minneapolis (MN): IPUMS; 2023. https://www.ipums.org/projects/ipums-nhgis. [cited 2025 Jan 09].

[72] Melendez R, Clarke P, Pan L, Li M, Khan A, Gomez-Lopez I, et al. National Neighborhood Data Archive (NaNDA): Land Cover by Census Tract and ZIP Code Tabulation Area, United States, 1985–2023. Ann Arbor (MI) : Inter-university Consortium for Political and Social Research [distributor]; 2025. 10.3886/ICPSR38598.v2. [cited 2025 Feb 01].

[73] ArcGIS Pro [computer program]. Version 3.5.2. Redlands (CA): Environmental Systems Research Institute, Inc.; 2025.

[74] RStudio [computer program]. Version 2025.5.0.496. Boston (MA): Posit Software, PBC; 2025.

[75] Brown C. Will Trump end the first federal heat protections for workers? New York Times [Internet]. 2025 Jan 22 [cited 2025 Feb 09]; Economy. https://www.nytimes.com/2025/06/16/climate/federal-workplace-heat-rules.html.

[76] New York State Department of Health. Occupational Health Surveillance Program 2019 [Available from: https://www.health.ny.gov/environmental/workplace/occupational_health_surveillance/.

[77] New Jersey Department of Health. Occupational Health Surveillance 2025 [Available from: https://www.nj.gov/health/workplacehealthandsafety/occupational-health-surveillance/.

[78] Gerstein T, Gong L. How local government can protect workers’ rights even when states do not want them to: opportunities for local creativity and persistence despite double preemption. Fordham Urb LJ. 2023;51:977.

[79] Kearl Z, Vogel J. Urban extreme heat, climate change, and saving lives: lessons from Washington state. Urban Climate. 2023;47:101392.

[80] Sheridan SC, Dolney TJ. Heat, mortality, and level of urbanization: measuring vulnerability across Ohio, USA. Climate Res. 2003;24:255–65.

[81] Barbeau EM, Krieger N, Soobader M-J. Working class matters: socioeconomic disadvantage, race/ethnicity, gender, and smoking in NHIS 2000. Am J Public Health. 2004;94(2):269–78. 14759942 10.2105/ajph.94.2.269 PMC1448243

[82] Syamlal G, Mazurek JM, Hendricks SA, Jamal A. Cigarette smoking trends among U.S. working adult by industry and occupation: findings from the 2004-2012 National Health Interview Survey. Nicotine Tob Res. 2015;17(5):599–606. 25239956 10.1093/ntr/ntu185 PMC4547354

[83] Buring JE, Evans DA, Fiore M, Rosner B, Hennekens CH. Occupation and risk of death from coronary heart disease. JAMA. 1987;258(6):791–2. 3613006

[84] Barnett E, Armstrong DL, Casper ML. Social class and premature mortality among men: a method for state-based surveillance. Am J Public Health. 1997;87(9):1521–5. 9314808 10.2105/ajph.87.9.1521 PMC1380982

[85] Mora C, Counsell CWW, Bielecki CR, Louis LV. Twenty-seven ways a heat wave can kill you: deadly heat in the era of climate change. Circ Cardiovasc Qual Outcomes. 2017;10(11). 10.1161/CIRCOUTCOMES.117.004233 29122837

[86] Gao C, Sanchez KM, Lovinsky-Desir S. Structural and social determinants of inequitable environmental exposures in the United States. Clin Chest Med. 2023. 10.1016/j.ccm.2023.03.002 37517826

[87] Chung-Bridges K, Muntaner C, Fleming LE, Lee DJ, Arheart KL, LeBlanc WG, et al. Occupational segregation as a determinant of US worker health. Am J Ind Med. 2008;51(8):555–67. 18553362 10.1002/ajim.20599

[88] Murray LR. Sick and tired of being sick and tired: scientific evidence, methods, and research implications for racial and ethnic disparities in occupational health. Am J Public Health. 2003;93(2):221–6. 12554573 10.2105/ajph.93.2.221 PMC1447720

[89] New York City Human Resources Administration (HRA). Cooling Assistance Benefit: Financial help to cover the cost and installation of an air conditioner or fan 2025 [Available from: https://access.nyc.gov/programs/cooling-assistance-benefit/.

[90] State of Oregon Bureau of Labor and Industries. Oregon sick leave and the Oregon Family and Medical Leave Act (OFLA) 2024 [Available from: https://www.oregon.gov/boli/workers/pages/sick-time.aspx.

[91] Madrigano J, Jack D, Anderson GB, Bell ML, Kinney PL. Temperature, ozone, and mortality in urban and non-urban counties in the northeastern United States. Environ Health. 2015;14:3. 25567355 10.1186/1476-069X-14-3 PMC4417233

[92] U.S. Environmental Protection Agency (EPA). Climate Change Indictors: Heat Wabes 2025 [Available from: https://www.epa.gov/climate-indicators/climate-change-indicators-heat-waves.

[93] Ierardi AM, Pavilonis B. New York City occupations at-risk of heat stress: integrating O*NET and BLS data for occupational insights. Ann Work Expo Health. 2025. 10.1093/annweh/wxaf022 40407046

[94] Gaffney A. Government Science Data May Soon Be Hidden. They’re Racing to Copy It. The New York times. 2025 2025.

[95] Davenport C. E.P.A. Workers Who Assist Heavily Polluted Communities Are Put on Leave. The New York times. 2025 2025.

[96] Schope J. New Jersey Heat Vulnerability Index Technical documentation available online at: https://njhazadapt.rutgers.edu/files/metadata/HVI_Methodology_Shope_12_06_2022_V2.pdf2022 [Available from: https://www.arcgis.com/apps/mapviewer/index.html?layers=a1e4b9530b7d4af4815470b52ece58e7.

[97] McCauslang College of Arts and Sciences, University of South Carolina. Social Vulnerability Index for the United States — 2010–14 and 2019 Indexes Available online at: https://www.sc.edu/study/colleges_schools/artsandsciences/centers_and_institutes/hvri/data_and_resources/sovi/: University of South Carolina,; 2024 [

[98] Tee Lewis PG, Chiu WA, Nasser E, Proville J, Barone A, Danforth C, et al. Characterizing vulnerabilities to climate change across the United States. Environ Int. 2023;172:107772. 36731185 10.1016/j.envint.2023.107772 PMC10214772

[99] New York City Department of Health. New York City PUMAs and Community Districts 2010 [Available from: https://www.nyc.gov/assets/planning/download/pdf/data-maps/nyc-population/census2010/puma_cd_map.pdf.

