# Supplementary material for: Occupational characteristics are missing from heat vulnerability indices: a study in New York and New Jersey
Source: Environ Health. 2026 Mar 14;25:38. doi: 10.1186/s12940-026-01284-w (PMC13101271; doi:10.1186/s12940-026-01284-w)
Supplement: Supplementary file 2 — Supplementary Material 2. [file 12940_2026_1284_MOESM2_ESM.docx]

**Occupational Characteristics Are Missing from Heat Vulnerability Indices: A study in New York and New Jersey**

**Author Names and Affiliations:**

Zoey Laskaris^a^, Sherry Baron^b^, Steven B. Markowitz^c^

^a^Barry Commoner Center for Health & the Environment, Queens College, City University of New York

65-30 Kissena Blvd, Flushing, NY 11367, USA

**Corresponding Author:** Zoey Laskaris ([zoey.laskaris@qc.cuny.edu](mailto:zoey.laskaris@qc.cuny.edu))

**Supplemental Materials**

Contents

[Supplemental Table 1- Distribution of occupational heat exposure risk scores by Standard Occupational Classification (SOC) Major Occupation Groups (2-digit SOC codes) 2](#_Toc204857225)

[Supplemental Table 2: Outdoor and indoor occupational heat exposure risk scores by detailed Standard Occupational Classification (SOC) occupation titles (6-digit codes) 4](#_Toc204857226)

[Supplemental Table 3 – Spearman’s correlation between outdoor and indoor occupational heat exposure and social, health, and environmental vulnerability factors in New York State stratified by urbanicity 32](#_Toc204857227)

[Supplemental Table 4– Spearman’s correlation between outdoor and indoor occupational heat exposure and social, health, and environmental vulnerability factors in New Jersey stratified by urbanicity 33](#_Toc204857228)

### Supplemental Table 1- Distribution of occupational heat exposure risk scores by Standard Occupational Classification (SOC) Major Occupation Groups (2-digit SOC codes)

|  | **Outdoor Heat (Exposed to Weather) Exposure Risk Score^1^** | | | **Outdoor Heat (Under Cover) Exposure Risk Score^1^** | | | **Indoor Heat (Not Environmentally Controlled) Exposure Risk Score^2^** | | |
| --- | --- | --- | --- | --- | --- | --- | --- | --- | --- |
| **Major Occupation Group (SOC Code)** | **Mean (SD)** | **Median** | **Range (min, max)** | **Mean (SD)** | **Median** | **Range (min, max)** | **Mean (SD)** | **Median** | **Range (min, max)** |
| Management Occupations(11-0000) | 29.6 (22.8) | 28.1 | 0.0, 94.5 | 19.1 (16.5) | 15.5 | 1.0, 74.5 | 25.8 (19.7) | 21.5 | 1.2, 79.5 |
| Business and Financial Operations Occupations(13-0000) | 19.0 (21.6) | 12.0 | 0.0, 79.2 | 12.1 (11.6) | 10.2 | 0.0, 48.2 | 17.4 (14.6) | 14.5 | 1.0, 50.0 |
| Computer and Mathematical Occupations(15-0000) | 6.7 (6.4) | 3.9 | 0.0, 24.8 | 5.6 (5.6) | 3.0 | 0.0, 21.2 | 9.9 (8.2) | 7.9 | 0.0, 29.4 |
| Architecture and Engineering Occupations(17-0000) | 35.2 (19.1) | 32.4 | 3.2, 77.6 | 24.8 (13.7) | 22.6 | 2.8, 60.0 | 37.9 (14.2) | 36.9 | 13.0, 62.8 |
| Life, Physical, and Social Science Occupations(19-0000) | 31.4 (25.8) | 23.1 | 0.5, 85.2 | 23.0 (19.8) | 16.6 | 0.0, 65.8 | 32.3 (23.2) | 26.1 | 2.5, 79.8 |
| Community and Social Service Occupations(21-0000) | 22.3 (16.4) | 21.4 | 4.5, 62.2 | 14.3 (8.1) | 14.5 | 1.2, 25.2 | 22.6 (11.5) | 23.6 | 3.2, 50.8 |
| Legal Occupations(23-0000) | 5.3 (4.1) | 5.5 | 0.0, 11.2 | 1.8 (2.2) | 0.5 | 0.0, 5.2 | 4.6 (5.2) | 3.2 | 0.0, 14.5 |
| Educational Instruction and Library Occupations(25-0000) | 16.8 (16.2) | 11.2 | 0.0, 60.5 | 10.1 (9.8) | 7.4 | 0.0, 53.0 | 18.1 (12.9) | 16.8 | 1.0, 66.8 |
| Arts, Design, Entertainment, Sports, and Media Occupations(27-0000) | 26.2 (18.8) | 20.6 | 0.0, 80.0 | 17.0 (11.4) | 16.5 | 0.0, 43.2 | 23.8 (14.2) | 22.6 | 0.8, 61.0 |
| Healthcare Practitioners and Technical Occupations(29-0000) | 6.3 (13.9) | 1.2 | 0.0, 79.5 | 4.4 (8.6) | 1.2 | 0.0, 45.5 | 7.7 (9.9) | 4.2 | 0.0, 60.5 |
| Healthcare Support Occupations(31-0000) | 11.9 (14.2) | 6.8 | 0.0, 44.8 | 8.6 (9.1) | 5.5 | 0.0, 27.5 | 8.7 (7.3) | 10.0 | 0.0, 23.0 |
| Protective Service Occupations(33-0000) | 67.2 (29.0) | 81.9 | 12.5, 99.8 | 34.2 (17.7) | 39.2 | 6.2, 58.8 | 46.7 (22.6) | 50.2 | 9.0, 87.5 |
| Food Preparation and Serving Related Occupations(35-0000) | 16.4 (12.5) | 16.5 | 1.2, 40.0 | 13.9 (10.3) | 11.2 | 0.0, 34.2 | 18.1 (12.5) | 13.0 | 5.0, 51.8 |
| Building and Grounds Cleaning and Maintenance Occupations(37-0000) | 70.8 (36.7) | 92.9 | 7.8, 99.5 | 23.3 (16.9) | 17.0 | 7.2, 52.2 | 37.0 (18.2) | 32.1 | 7.8, 61.0 |
| Personal Care and Service Occupations(39-0000) | 34.0 (29.3) | 29.0 | 0.0, 94.5 | 22.3 (20.8) | 17.2 | 0.0, 62.5 | 22.8 (17.7) | 18.0 | 0.0, 54.2 |
| Sales and Related Occupations(41-0000) | 29.6 (21.2) | 32.0 | 1.5, 71.8 | 16.4 (11.5) | 15.2 | 0.0, 48.8 | 21.9 (18.2) | 18.2 | 1.5, 84.5 |
| Office and Administrative Support Occupations(43-0000) | 13.4 (21.5) | 6.9 | 0.0, 100.0 | 4.8 (6.3) | 2.5 | 0.0, 32.8 | 13.3 (17.4) | 5.2 | 0.0, 88.8 |
| Farming, Fishing, and Forestry Occupations(45-0000) | 80.9 (22.0) | 84.4 | 16.2, 99.0 | 43.4 (19.3) | 47.5 | 7.5, 68.5 | 47.1 (19.9) | 51.4 | 16.2, 75.5 |
| Construction and Extraction Occupations(47-0000) | 75.0 (25.4) | 83.8 | 19.0, 100.0 | 41.1 (15.0) | 40.1 | 11.8, 70.8 | 56.3 (15.1) | 56.6 | 24.0, 89.5 |
| Installation, Maintenance, and Repair Occupations(49-0000) | 64.1 (26.4) | 71.5 | 0.0, 100.0 | 39.5 (19.2) | 37.2 | 0.0, 86.0 | 66.1 (22.0) | 73.1 | 0.0, 98.5 |
| Production Occupations(51-0000) | 20.2 (22.3) | 12.8 | 0.0, 96.0 | 12.9 (17.2) | 6.8 | 0.0, 79.2 | 51.1 (26.0) | 56.8 | 2.0, 99.0 |
| Transportation and Material Moving Occupations(53-0000) | 71.9 (23.7) | 78.8 | 3.2, 100.0 | 38.7 (19.5) | 38.8 | 3.5, 76.0 | 51.9 (20.6) | 53.0 | 6.0, 86.5 |

^1^ Outdoor heat exposure risk scores were derived from two ONET Work Context Survey questions: “How often does your current job require you to work outdoors, exposed to all weather conditions?” and “How often does your current job require you to work outdoors, under cover (like in an open shed)?” Responses were on a 5-point frequency scale, converted to 0–100 (0 = Never, 25 = Once a year or more but not every month, 50 = Once a month or more but not every week, 75 = Once a week or more but not every day, 100 = Every day); scores ≥75 were classified as elevated risk.
^2^ Indoor heat exposure risk was based on the O*NET question: “How often does your current job require you to work indoors in an environment that is not environmentally controlled (like a warehouse without air conditioning)?” and scored using the same method.

### Supplemental Table 2: Outdoor and indoor occupational heat exposure risk scores by detailed Standard Occupational Classification (SOC) occupation titles (6-digit codes)

| **Occupation** | | **Outdoor Heat Exposure Risk Score^1^** | | **Indoor Heat Exposure Risk Score^2^** |
| --- | --- | --- | --- | --- |
| **Major Occupation Group (SOC Code)^3^** | **Occupation (SOC Code)** | **Outdoors - exposed to weather** | **Outdoors - under cover** | **Indoors - not environmentally controlled** |
| Management Occupations (11-0000) | Chief Executives (11-1011) | 29.4 | 23.0 | 29.2 |
|  | General and Operations Managers (11-1021) | 48.2 | 19.8 | 28.0 |
|  | Advertising and Promotions Managers (11-2011) | 36.5 | 25.0 | 17.0 |
|  | Marketing Managers (11-2021) | 9.5 | 6.2 | 21.5 |
|  | Sales Managers (11-2022) | 29.2 | 24.0 | 22.8 |
|  | Administrative Services Managers (11-3012) | 19.8 | 17.2 | 22.5 |
|  | Facilities Managers (11-3013) | 41.5 | 27.5 | 46.2 |
|  | Computer and Information Systems Managers (11-3021) | 4.2 | 3.2 | 16.5 |
|  | Financial Managers (11-3031) | 4.8 | 3.4 | 2.2 |
|  | Industrial Production Managers (11-3051) | 56.2 | 38.6 | 71.0 |
|  | Purchasing Managers (11-3061) | 18.5 | 14.5 | 22.2 |
|  | Transportation, Storage, and Distribution Managers (11-3071) | 35.9 | 22.6 | 58.8 |
|  | Compensation and Benefits Managers (11-3111) | 1.2 | 1.2 | 1.2 |
|  | Human Resources Managers (11-3121) | 7.2 | 8.2 | 19.8 |
|  | Training and Development Managers (11-3131) | 6.2 | 8.0 | 8.0 |
|  | Farmers, Ranchers, and Other Agricultural Managers (11-9013) | **94.5** | 74.5 | **79.5** |
|  | Construction Managers (11-9021) | **77.5** | 55.0 | 55.0 |
|  | Education and Childcare Administrators, Preschool and Daycare (11-9031) | 32.5 | 9.8 | 2.8 |
|  | Education Administrators, Kindergarten through Secondary (11-9032) | 44.5 | 24.0 | 13.2 |
|  | Education Administrators, Postsecondary (11-9033) | 16.0 | 6.8 | 12.5 |
|  | Architectural and Engineering Managers (11-9041) | 28.1 | 24.4 | 40.0 |
|  | Food Service Managers (11-9051) | 18.0 | 15.5 | 13.8 |
|  | Gambling Managers (11-9071) | 11.0 | 8.0 | 11.5 |
|  | Lodging Managers (11-9081) | 11.2 | 1.8 | 6.0 |
|  | Medical and Health Services Managers (11-9111) | 0.0 | 1.0 | 7.2 |
|  | Natural Sciences Managers (11-9121) | 19.7 | 13.7 | 18.6 |
|  | Postmasters and Mail Superintendents (11-9131) | 46.0 | 5.5 | 31.0 |
|  | Property, Real Estate, and Community Association Managers (11-9141) | 51.2 | 26.2 | 40.8 |
|  | Social and Community Service Managers (11-9151) | 17.0 | 7.0 | 7.5 |
|  | Emergency Management Directors (11-9161) | 46.5 | 34.0 | 38.8 |
|  | Funeral Home Managers (11-9171) | 67.0 | 47.2 | 39.5 |
|  | Personal Service Managers, All Other (11-9179) | 13.9 | 9.5 | 11.2 |
|  | Managers, All Other (11-9199) | 35.0 | 25.1 | 35.0 |
| Business and Financial Operations Occupations (13-0000) | Agents and Business Managers of Artists, Performers, and Athletes (13-1011) | 4.5 | 4.5 | 4.0 |
|  | Buyers and Purchasing Agents, Farm Products (13-1021) | 30.0 | 25.5 | 44.8 |
|  | Wholesale and Retail Buyers, Except Farm Products (13-1022) | 8.8 | 5.0 | 30.2 |
|  | Purchasing Agents, Except Wholesale, Retail, and Farm Products (13-1023) | 15.2 | 21.8 | 26.0 |
|  | Claims Adjusters, Examiners, and Investigators (13-1031) | 16.8 | 7.2 | 6.8 |
|  | Insurance Appraisers, Auto Damage (13-1032) | **78.2** | 39.2 | 50.0 |
|  | Compliance Officers (13-1041) | 43.3 | 28.6 | 38.4 |
|  | Cost Estimators (13-1051) | 13.0 | 10.2 | 17.0 |
|  | Human Resources Specialists (13-1071) | 11.5 | 10.5 | 3.5 |
|  | Farm Labor Contractors (13-1074) | **79.2** | 48.2 | 48.5 |
|  | Labor Relations Specialists (13-1075) | 10.5 | 10.8 | 20.2 |
|  | Logisticians (13-1081) | 24.1 | 21.9 | 35.1 |
|  | Management Analysts (13-1111) | 18.8 | 13.0 | 20.0 |
|  | Meeting, Convention, and Event Planners (13-1121) | 33.0 | 23.2 | 9.5 |
|  | Fundraisers (13-1131) | 19.0 | 15.5 | 20.2 |
|  | Compensation, Benefits, and Job Analysis Specialists (13-1141) | 6.0 | 6.0 | 6.2 |
|  | Training and Development Specialists (13-1151) | 12.0 | 13.0 | 17.5 |
|  | Market Research Analysts and Marketing Specialists (13-1161) | 5.9 | 2.1 | 4.9 |
|  | Business Operations Specialists, All Other (13-1199) | 16.7 | 11.8 | 20.6 |
|  | Accountants and Auditors (13-2011) | 6.8 | 6.2 | 9.5 |
|  | Appraisers and Assessors of Real Estate (13-2023) | 72.0 | 13.5 | 28.2 |
|  | Budget Analysts (13-2031) | 3.8 | 0.0 | 3.5 |
|  | Credit Analysts (13-2041) | 4.0 | 1.2 | 1.2 |
|  | Personal Financial Advisors (13-2052) | 2.0 | 2.0 | 3.0 |
|  | Insurance Underwriters (13-2053) | 5.0 | 3.2 | 5.8 |
|  | Financial Examiners (13-2061) | 0.0 | 0.0 | 1.0 |
|  | Credit Counselors (13-2071) | 9.0 | 6.0 | 5.2 |
|  | Loan Officers (13-2072) | 23.0 | 10.2 | 28.8 |
|  | Tax Examiners and Collectors, and Revenue Agents (13-2081) | 3.0 | 1.2 | 12.2 |
|  | Tax Preparers (13-2082) | 2.2 | 1.2 | 4.5 |
|  | Financial Specialists, All Other (13-2099) | 12.0 | 10.5 | 14.5 |
| Computer and Mathematical Occupations (15-0000) | Computer Systems Analysts (15-1211) | 4.0 | 5.0 | 5.4 |
|  | Information Security Analysts (15-1212) | 13.0 | 15.5 | 17.0 |
|  | Computer and Information Research Scientists (15-1221) | 1.5 | 1.0 | 10.0 |
|  | Computer Network Support Specialists (15-1231) | 12.8 | 11.2 | 25.5 |
|  | Computer User Support Specialists (15-1232) | 15.8 | 9.5 | 12.5 |
|  | Computer Network Architects (15-1241) | 24.8 | 21.2 | 29.4 |
|  | Database Administrators (15-1242) | 3.0 | 3.0 | 8.8 |
|  | Database Architects (15-1243) | 1.2 | 0.6 | 3.5 |
|  | Network and Computer Systems Administrators (15-1244) | 10.8 | 3.0 | 17.0 |
|  | Computer Programmers (15-1251) | 4.8 | 4.5 | 0.8 |
|  | Software Quality Assurance Analysts and Testers (15-1253) | 3.0 | 3.0 | 7.2 |
|  | Web Developers (15-1254) | 1.0 | 2.0 | 2.0 |
|  | Web and Digital Interface Designers (15-1255) | 3.8 | 2.5 | 2.5 |
|  | Computer Occupations, All Other (15-1299) | 12.7 | 9.2 | 16.6 |
|  | Actuaries (15-2011) | 0.0 | 0.0 | 0.0 |
|  | Mathematicians (15-2021) | 5.2 | 3.0 | 5.2 |
|  | Operations Research Analysts (15-2031) | 9.5 | 9.5 | 16.8 |
|  | Statisticians (15-2041) | 2.4 | 2.2 | 6.0 |
|  | Data Scientists (15-2051) | 1.2 | 0.0 | 2.9 |
|  | Mathematical Science Occupations, All Other (15-2099) | 3.8 | 6.0 | 8.5 |
| Architecture and Engineering Occupations (17-0000) | Architects, Except Landscape and Naval (17-1011) | 41.2 | 37.0 | 35.8 |
|  | Landscape Architects (17-1012) | 50.0 | 29.5 | 29.5 |
|  | Cartographers and Photogrammetrists (17-1021) | 25.5 | 5.2 | 13.0 |
|  | Surveyors (17-1022) | **77.6** | 28.9 | 53.1 |
|  | Aerospace Engineers (17-2011) | 14.0 | 19.0 | 42.2 |
|  | Agricultural Engineers (17-2021) | 62.5 | 60.0 | 58.8 |
|  | Bioengineers and Biomedical Engineers (17-2031) | 8.8 | 9.8 | 14.2 |
|  | Chemical Engineers (17-2041) | 30.8 | 39.2 | 50.0 |
|  | Civil Engineers (17-2051) | 46.1 | 25.8 | 36.8 |
|  | Computer Hardware Engineers (17-2061) | 9.2 | 7.5 | 13.0 |
|  | Electrical Engineers (17-2071) | 30.2 | 21.5 | 35.8 |
|  | Electronics Engineers, Except Computer (17-2072) | 8.2 | 7.0 | 30.6 |
|  | Environmental Engineers (17-2081) | 55.0 | 45.2 | 53.5 |
|  | Health and Safety Engineers, Except Mining Safety Engineers and Inspectors (17-2111) | 48.4 | 39.8 | 53.2 |
|  | Industrial Engineers (17-2112) | 21.9 | 20.1 | 43.1 |
|  | Marine Engineers and Naval Architects (17-2121) | 52.0 | 47.2 | 62.8 |
|  | Materials Engineers (17-2131) | 16.8 | 19.0 | 37.0 |
|  | Mechanical Engineers (17-2141) | 22.5 | 23.7 | 29.8 |
|  | Mining and Geological Engineers, Including Mining Safety Engineers (17-2151) | 46.8 | 28.2 | 55.2 |
|  | Nuclear Engineers (17-2161) | 27.5 | 23.8 | 38.0 |
|  | Petroleum Engineers (17-2171) | 30.2 | 17.5 | 27.8 |
|  | Engineers, All Other (17-2199) | 25.6 | 19.3 | 34.2 |
|  | Architectural and Civil Drafters (17-3011) | 34.0 | 18.2 | 19.8 |
|  | Electrical and Electronics Drafters (17-3012) | 27.8 | 11.8 | 24.0 |
|  | Mechanical Drafters (17-3013) | 3.2 | 2.8 | 16.5 |
|  | Aerospace Engineering and Operations Technologists and Technicians (17-3021) | 18.0 | 12.2 | 24.2 |
|  | Civil Engineering Technologists and Technicians (17-3022) | 63.0 | 15.5 | 37.8 |
|  | Electrical and Electronic Engineering Technologists and Technicians (17-3023) | 36.2 | 30.2 | 42.2 |
|  | Electro-Mechanical and Mechatronics Technologists and Technicians (17-3024) | 37.4 | 30.5 | 59.9 |
|  | Environmental Engineering Technologists and Technicians (17-3025) | 68.2 | 37.5 | 48.2 |
|  | Industrial Engineering Technologists and Technicians (17-3026) | 11.2 | 10.4 | 29.5 |
|  | Mechanical Engineering Technologists and Technicians (17-3027) | 37.1 | 32.0 | 51.2 |
|  | Engineering Technologists and Technicians, Except Drafters, All Other (17-3029) | 50.5 | 47.8 | 54.5 |
|  | Surveying and Mapping Technicians (17-3031) | 57.8 | 19.0 | 33.0 |
| Life, Physical, and Social Science Occupations (19-0000) | Animal Scientists (19-1011) | 56.5 | 59.8 | 60.8 |
|  | Food Scientists and Technologists (19-1012) | 18.2 | 15.2 | 28.5 |
|  | Soil and Plant Scientists (19-1013) | 72.5 | 51.2 | 67.5 |
|  | Biochemists and Biophysicists (19-1021) | 1.2 | 4.0 | 11.2 |
|  | Microbiologists (19-1022) | 20.5 | 18.2 | 25.0 |
|  | Zoologists and Wildlife Biologists (19-1023) | 59.8 | 41.8 | 50.5 |
|  | Biological Scientists, All Other (19-1029) | 19.6 | 14.5 | 21.8 |
|  | Conservation Scientists (19-1031) | 72.2 | 49.8 | 56.4 |
|  | Foresters (19-1032) | **76.2** | 35.5 | 55.2 |
|  | Epidemiologists (19-1041) | 15.8 | 10.5 | 25.0 |
|  | Medical Scientists, Except Epidemiologists (19-1042) | 3.5 | 1.8 | 4.0 |
|  | Astronomers (19-2011) | 17.5 | 16.2 | 19.5 |
|  | Physicists (19-2012) | 2.8 | 3.5 | 5.0 |
|  | Atmospheric and Space Scientists (19-2021) | 30.0 | 21.2 | 20.0 |
|  | Chemists (19-2031) | 3.2 | 3.2 | 15.2 |
|  | Materials Scientists (19-2032) | 17.8 | 12.5 | 33.2 |
|  | Environmental Scientists and Specialists, Including Health (19-2041) | 33.8 | 21.3 | 26.1 |
|  | Geoscientists, Except Hydrologists and Geographers (19-2042) | 50.8 | 35.2 | 35.2 |
|  | Hydrologists (19-2043) | 52.2 | 32.5 | 39.2 |
|  | Physical Scientists, All Other (19-2099) | 31.5 | 17.0 | 14.0 |
|  | Economists (19-3011) | 5.6 | 3.9 | 3.8 |
|  | Survey Researchers (19-3022) | 7.2 | 0.0 | 4.2 |
|  | Industrial-Organizational Psychologists (19-3032) | 5.0 | 7.8 | 12.5 |
|  | Clinical and Counseling Psychologists (19-3033) | 8.2 | 9.5 | 13.0 |
|  | School Psychologists (19-3034) | 9.5 | 4.8 | 4.0 |
|  | Psychologists, All Other (19-3039) | 0.5 | 0.0 | 5.6 |
|  | Sociologists (19-3041) | 5.2 | 5.0 | 2.5 |
|  | Urban and Regional Planners (19-3051) | 31.0 | 11.0 | 23.0 |
|  | Anthropologists and Archeologists (19-3091) | 35.5 | 28.8 | 37.5 |
|  | Geographers (19-3092) | 30.0 | 22.5 | 28.8 |
|  | Historians (19-3093) | 30.2 | 23.0 | 45.0 |
|  | Political Scientists (19-3094) | 4.0 | 3.8 | 9.5 |
|  | Social Scientists and Related Workers, All Other (19-3099) | 23.8 | 11.8 | 20.0 |
|  | Agricultural Technicians (19-4012) | **77.4** | 61.1 | 66.1 |
|  | Food Science Technicians (19-4013) | 7.2 | 3.2 | 60.2 |
|  | Biological Technicians (19-4021) | 11.5 | 10.5 | 12.8 |
|  | Chemical Technicians (19-4031) | 22.5 | 26.8 | 40.8 |
|  | Environmental Science and Protection Technicians, Including Health (19-4042) | 64.8 | 54.5 | 63.8 |
|  | Geological Technicians, Except Hydrologic Technicians (19-4043) | 37.5 | 24.0 | 38.5 |
|  | Nuclear Technicians (19-4051) | 65.4 | 49.0 | **77.2** |
|  | Social Science Research Assistants (19-4061) | 5.5 | 3.0 | 4.2 |
|  | Forest and Conservation Technicians (19-4071) | **85.2** | 56.8 | **79.8** |
|  | Forensic Science Technicians (19-4092) | 73.2 | 65.8 | 62.0 |
|  | Life, Physical, and Social Science Technicians, All Other (19-4099) | 21.9 | 6.8 | 26.1 |
|  | Occupational Health and Safety Specialists (19-5011) | 58.8 | 45.8 | 64.2 |
|  | Occupational Health and Safety Technicians (19-5012) | 62.5 | 52.5 | 69.0 |
| Community and Social Service Occupations (21-0000) | Substance Abuse and Behavioral Disorder Counselors (21-1011) | 6.2 | 1.2 | 3.2 |
|  | Educational, Guidance, and Career Counselors and Advisors (21-1012) | 8.0 | 9.8 | 8.2 |
|  | Marriage and Family Therapists (21-1013) | 4.5 | 7.0 | 25.5 |
|  | Mental Health Counselors (21-1014) | 6.5 | 4.8 | 10.5 |
|  | Rehabilitation Counselors (21-1015) | 24.5 | 21.0 | 29.8 |
|  | Child, Family, and School Social Workers (21-1021) | 45.8 | 24.0 | 23.5 |
|  | Healthcare Social Workers (21-1022) | 18.2 | 8.8 | 27.8 |
|  | Mental Health and Substance Abuse Social Workers (21-1023) | 26.0 | 11.2 | 26.0 |
|  | Health Education Specialists (21-1091) | 16.5 | 18.8 | 29.8 |
|  | Probation Officers and Correctional Treatment Specialists (21-1092) | 62.2 | 23.2 | 50.8 |
|  | Social and Human Service Assistants (21-1093) | 32.2 | 21.0 | 22.0 |
|  | Community Health Workers (21-1094) | 25.8 | 25.2 | 18.2 |
|  | Clergy (21-2011) | 24.8 | 17.8 | 23.8 |
|  | Directors, Religious Activities and Education (21-2021) | 10.5 | 7.0 | 18.0 |
| Legal Occupations (23-0000) | Lawyers (23-1011) | 5.5 | 2.5 | 5.5 |
|  | Judicial Law Clerks (23-1012) | 0.0 | 0.0 | 0.0 |
|  | Administrative Law Judges, Adjudicators, and Hearing Officers (23-1021) | 0.2 | 0.2 | 1.2 |
|  | Arbitrators, Mediators, and Conciliators (23-1022) | 5.0 | 4.0 | 14.5 |
|  | Judges, Magistrate Judges, and Magistrates (23-1023) | 7.8 | 5.2 | 7.8 |
|  | Paralegals and Legal Assistants (23-2011) | 7.2 | 0.5 | 3.2 |
|  | Title Examiners, Abstractors, and Searchers (23-2093) | 11.2 | 0.0 | 0.0 |
| Educational Instruction and Library Occupations (25-0000) | Business Teachers, Postsecondary (25-1011) | 5.5 | 6.0 | 13.5 |
|  | Computer Science Teachers, Postsecondary (25-1021) | 3.2 | 5.0 | 4.0 |
|  | Mathematical Science Teachers, Postsecondary (25-1022) | 0.5 | 0.8 | 5.2 |
|  | Architecture Teachers, Postsecondary (25-1031) | 24.2 | 7.2 | 26.8 |
|  | Engineering Teachers, Postsecondary (25-1032) | 8.0 | 6.0 | 33.0 |
|  | Agricultural Sciences Teachers, Postsecondary (25-1041) | 37.2 | 11.5 | 29.5 |
|  | Biological Science Teachers, Postsecondary (25-1042) | 21.5 | 11.5 | 16.2 |
|  | Forestry and Conservation Science Teachers, Postsecondary (25-1043) | 55.0 | 25.2 | 40.0 |
|  | Atmospheric, Earth, Marine, and Space Sciences Teachers, Postsecondary (25-1051) | 26.8 | 15.0 | 19.5 |
|  | Chemistry Teachers, Postsecondary (25-1052) | 2.2 | 2.0 | 2.8 |
|  | Environmental Science Teachers, Postsecondary (25-1053) | 28.8 | 21.5 | 23.5 |
|  | Physics Teachers, Postsecondary (25-1054) | 8.5 | 4.8 | 20.5 |
|  | Anthropology and Archeology Teachers, Postsecondary (25-1061) | 22.0 | 9.8 | 16.5 |
|  | Area, Ethnic, and Cultural Studies Teachers, Postsecondary (25-1062) | 6.8 | 5.0 | 14.5 |
|  | Economics Teachers, Postsecondary (25-1063) | 2.0 | 1.2 | 1.0 |
|  | Geography Teachers, Postsecondary (25-1064) | 22.8 | 11.8 | 23.5 |
|  | Political Science Teachers, Postsecondary (25-1065) | 2.2 | 1.8 | 6.2 |
|  | Psychology Teachers, Postsecondary (25-1066) | 0.2 | 0.2 | 2.5 |
|  | Sociology Teachers, Postsecondary (25-1067) | 3.8 | 3.5 | 3.5 |
|  | Health Specialties Teachers, Postsecondary (25-1071) | 7.8 | 4.2 | 6.8 |
|  | Nursing Instructors and Teachers, Postsecondary (25-1072) | 5.8 | 6.8 | 6.8 |
|  | Education Teachers, Postsecondary (25-1081) | 1.5 | 1.5 | 15.8 |
|  | Library Science Teachers, Postsecondary (25-1082) | 3.2 | 0.8 | 3.2 |
|  | Criminal Justice and Law Enforcement Teachers, Postsecondary (25-1111) | 13.2 | 10.0 | 17.8 |
|  | Law Teachers, Postsecondary (25-1112) | 3.8 | 5.0 | 4.5 |
|  | Social Work Teachers, Postsecondary (25-1113) | 14.5 | 7.5 | 17.0 |
|  | Art, Drama, and Music Teachers, Postsecondary (25-1121) | 8.0 | 7.0 | 11.2 |
|  | Communications Teachers, Postsecondary (25-1122) | 4.2 | 3.0 | 10.0 |
|  | English Language and Literature Teachers, Postsecondary (25-1123) | 0.2 | 0.8 | 11.2 |
|  | Foreign Language and Literature Teachers, Postsecondary (25-1124) | 0.0 | 0.0 | 5.5 |
|  | History Teachers, Postsecondary (25-1125) | 0.2 | 0.2 | 20.5 |
|  | Philosophy and Religion Teachers, Postsecondary (25-1126) | 0.0 | 0.0 | 2.8 |
|  | Family and Consumer Sciences Teachers, Postsecondary (25-1192) | 4.8 | 1.2 | 10.2 |
|  | Recreation and Fitness Studies Teachers, Postsecondary (25-1193) | 27.2 | 19.0 | 29.8 |
|  | Career/Technical Education Teachers, Postsecondary (25-1194) | 9.5 | 10.0 | 23.8 |
|  | Preschool Teachers, Except Special Education (25-2011) | 52.8 | 20.5 | 12.2 |
|  | Kindergarten Teachers, Except Special Education (25-2012) | 37.2 | 25.5 | 24.5 |
|  | Elementary School Teachers, Except Special Education (25-2021) | 38.0 | 1.2 | 17.0 |
|  | Middle School Teachers, Except Special and Career/Technical Education (25-2022) | 36.2 | 21.2 | 29.2 |
|  | Career/Technical Education Teachers, Middle School (25-2023) | 21.2 | 15.2 | 17.8 |
|  | Secondary School Teachers, Except Special and Career/Technical Education (25-2031) | 22.0 | 8.2 | 27.5 |
|  | Career/Technical Education Teachers, Secondary School (25-2032) | 19.5 | 22.0 | 34.0 |
|  | Special Education Teachers, Preschool (25-2051) | 45.8 | 23.8 | 6.8 |
|  | Special Education Teachers, Middle School (25-2057) | 19.2 | 15.5 | 21.8 |
|  | Special Education Teachers, Secondary School (25-2058) | 14.2 | 9.0 | 12.8 |
|  | Special Education Teachers, All Other (25-2059) | 53.8 | 33.2 | 56.0 |
|  | Adult Basic Education, Adult Secondary Education, and English as a Second Language Instructors (25-3011) | 4.8 | 4.8 | 15.0 |
|  | Self-Enrichment Teachers (25-3021) | 18.0 | 10.2 | 18.2 |
|  | Tutors (25-3041) | 2.5 | 3.5 | 4.5 |
|  | Archivists (25-4011) | 6.2 | 9.2 | 25.0 |
|  | Curators (25-4012) | 25.8 | 14.5 | 33.5 |
|  | Museum Technicians and Conservators (25-4013) | 23.0 | 19.2 | 36.5 |
|  | Librarians and Media Collections Specialists (25-4022) | 10.0 | 9.8 | 18.2 |
|  | Library Technicians (25-4031) | 12.5 | 6.8 | 16.0 |
|  | Farm and Home Management Educators (25-9021) | 60.5 | 53.0 | 66.8 |
|  | Instructional Coordinators (25-9031) | 20.2 | 8.2 | 9.8 |
|  | Teaching Assistants, Preschool, Elementary, Middle, and Secondary School, Except Special Education (25-9042) | 45.2 | 21.5 | 27.8 |
|  | Teaching Assistants, Postsecondary (25-9044) | 2.5 | 1.5 | 18.5 |
| Arts, Design, Entertainment, Sports, and Media Occupations (27-0000) | Art Directors (27-1011) | 5.5 | 6.0 | 16.2 |
|  | Craft Artists (27-1012) | 29.0 | 41.0 | 61.0 |
|  | Fine Artists, Including Painters, Sculptors, and Illustrators (27-1013) | 21.8 | 18.2 | 0.8 |
|  | Special Effects Artists and Animators (27-1014) | 15.2 | 7.2 | 3.2 |
|  | Commercial and Industrial Designers (27-1021) | 11.5 | 6.8 | 34.8 |
|  | Fashion Designers (27-1022) | 14.0 | 12.5 | 18.8 |
|  | Floral Designers (27-1023) | 57.0 | 4.2 | 29.5 |
|  | Graphic Designers (27-1024) | 9.2 | 8.8 | 10.5 |
|  | Interior Designers (27-1025) | 19.8 | 23.0 | 28.0 |
|  | Merchandise Displayers and Window Trimmers (27-1026) | 17.8 | 10.0 | 23.0 |
|  | Set and Exhibit Designers (27-1027) | 25.0 | 22.5 | 32.2 |
|  | Actors (27-2011) | 19.0 | 16.0 | 23.0 |
|  | Producers and Directors (27-2012) | 21.6 | 14.3 | 14.0 |
|  | Athletes and Sports Competitors (27-2021) | **80.0** | 41.5 | 50.8 |
|  | Coaches and Scouts (27-2022) | 56.5 | 6.5 | 34.2 |
|  | Umpires, Referees, and Other Sports Officials (27-2023) | 61.2 | 20.0 | 41.8 |
|  | Dancers (27-2031) | 18.0 | 18.5 | 22.2 |
|  | Choreographers (27-2032) | 19.0 | 23.8 | 31.0 |
|  | Music Directors and Composers (27-2041) | 12.5 | 7.2 | 13.8 |
|  | Musicians and Singers (27-2042) | 9.2 | 17.0 | 18.2 |
|  | Broadcast Announcers and Radio Disc Jockeys (27-3011) | 33.8 | 20.5 | 23.5 |
|  | News Analysts, Reporters, and Journalists (27-3023) | 41.5 | 29.2 | 35.2 |
|  | Public Relations Specialists (27-3031) | 21.5 | 21.5 | 18.2 |
|  | Editors (27-3041) | 15.8 | 7.5 | 1.2 |
|  | Technical Writers (27-3042) | 3.8 | 3.8 | 16.5 |
|  | Writers and Authors (27-3043) | 17.8 | 4.5 | 17.8 |
|  | Interpreters and Translators (27-3091) | 23.0 | 9.0 | 7.8 |
|  | Court Reporters and Simultaneous Captioners (27-3092) | 0.0 | 0.0 | 1.0 |
|  | Audio and Video Technicians (27-4011) | 10.8 | 9.5 | 19.8 |
|  | Broadcast Technicians (27-4012) | 35.8 | 24.5 | 25.5 |
|  | Sound Engineering Technicians (27-4014) | 28.0 | 32.5 | 30.5 |
|  | Photographers (27-4021) | 56.8 | 43.2 | 44.2 |
|  | Camera Operators, Television, Video, and Film (27-4031) | 52.5 | 30.8 | 41.0 |
|  | Film and Video Editors (27-4032) | 26.8 | 17.2 | 19.0 |
| Healthcare Practitioners and Technical Occupations (29-0000) | Chiropractors (29-1011) | 2.2 | 1.8 | 3.5 |
|  | Dentists, General (29-1021) | 1.8 | 1.2 | 20.8 |
|  | Oral and Maxillofacial Surgeons (29-1022) | 0.0 | 0.0 | 10.2 |
|  | Orthodontists (29-1023) | 0.5 | 4.2 | 1.2 |
|  | Prosthodontists (29-1024) | 0.0 | 0.0 | 0.0 |
|  | Dietitians and Nutritionists (29-1031) | 4.2 | 6.2 | 8.2 |
|  | Optometrists (29-1041) | 0.0 | 3.5 | 2.5 |
|  | Pharmacists (29-1051) | 0.8 | 0.8 | 0.5 |
|  | Physician Assistants (29-1071) | 4.8 | 3.0 | 6.4 |
|  | Podiatrists (29-1081) | 2.5 | 0.2 | 15.8 |
|  | Occupational Therapists (29-1122) | 47.8 | 23.1 | 29.8 |
|  | Physical Therapists (29-1123) | 8.5 | 2.8 | 4.0 |
|  | Radiation Therapists (29-1124) | 1.2 | 1.2 | 1.5 |
|  | Recreational Therapists (29-1125) | 46.0 | 37.8 | 31.5 |
|  | Respiratory Therapists (29-1126) | 1.2 | 0.2 | 5.8 |
|  | Speech-Language Pathologists (29-1127) | 9.2 | 0.0 | 13.5 |
|  | Exercise Physiologists (29-1128) | 25.0 | 18.2 | 19.2 |
|  | Therapists, All Other (29-1129) | 11.6 | 14.8 | 16.6 |
|  | Veterinarians (29-1131) | 13.8 | 14.0 | 12.5 |
|  | Registered Nurses (29-1141) | 3.1 | 5.0 | 5.2 |
|  | Nurse Anesthetists (29-1151) | 4.8 | 2.8 | 12.0 |
|  | Nurse Midwives (29-1161) | 1.5 | 0.8 | 4.5 |
|  | Nurse Practitioners (29-1171) | 7.5 | 8.8 | 13.0 |
|  | Audiologists (29-1181) | 0.0 | 1.2 | 6.2 |
|  | Anesthesiologists (29-1211) | 0.2 | 0.2 | 5.0 |
|  | Dermatologists (29-1213) | 0.0 | 1.8 | 2.0 |
|  | Family Medicine Physicians (29-1215) | 0.0 | 0.0 | 0.0 |
|  | General Internal Medicine Physicians (29-1216) | 0.0 | 0.0 | 3.5 |
|  | Neurologists (29-1217) | 2.0 | 0.0 | 1.2 |
|  | Obstetricians and Gynecologists (29-1218) | 0.0 | 0.0 | 0.5 |
|  | Pediatricians, General (29-1221) | 0.0 | 0.0 | 0.0 |
|  | Physicians, Pathologists (29-1222) | 0.2 | 0.2 | 0.2 |
|  | Psychiatrists (29-1223) | 1.0 | 0.5 | 1.0 |
|  | Radiologists (29-1224) | 0.0 | 0.0 | 1.0 |
|  | Physicians, All Other (29-1229) | 10.4 | 5.9 | 13.2 |
|  | Ophthalmologists, Except Pediatric (29-1241) | 0.0 | 0.0 | 0.0 |
|  | Acupuncturists (29-1291) | 0.0 | 0.0 | 12.2 |
|  | Dental Hygienists (29-1292) | 0.0 | 0.0 | 5.2 |
|  | Healthcare Diagnosing or Treating Practitioners, All Other (29-1299) | 1.0 | 0.5 | 1.0 |
|  | Medical and Clinical Laboratory Technologists (29-2011) | 0.4 | 0.4 | 1.8 |
|  | Medical and Clinical Laboratory Technicians (29-2012) | 1.0 | 0.0 | 2.8 |
|  | Cardiovascular Technologists and Technicians (29-2031) | 0.0 | 0.0 | 0.0 |
|  | Diagnostic Medical Sonographers (29-2032) | 1.2 | 0.0 | 4.2 |
|  | Nuclear Medicine Technologists (29-2033) | 0.5 | 2.5 | 0.5 |
|  | Radiologic Technologists and Technicians (29-2034) | 1.2 | 1.2 | 0.5 |
|  | Magnetic Resonance Imaging Technologists (29-2035) | 6.5 | 8.8 | 9.8 |
|  | Medical Dosimetrists (29-2036) | 0.0 | 0.0 | 1.2 |
|  | Dietetic Technicians (29-2051) | 4.2 | 6.0 | 15.8 |
|  | Pharmacy Technicians (29-2052) | 1.2 | 1.5 | 2.8 |
|  | Psychiatric Technicians (29-2053) | 38.0 | 22.0 | 14.2 |
|  | Surgical Technologists (29-2055) | 0.2 | 3.0 | 3.5 |
|  | Veterinary Technologists and Technicians (29-2056) | 25.8 | 6.2 | 10.2 |
|  | Ophthalmic Medical Technicians (29-2057) | 0.0 | 1.2 | 2.2 |
|  | Licensed Practical and Licensed Vocational Nurses (29-2061) | 4.0 | 0.5 | 6.2 |
|  | Opticians, Dispensing (29-2081) | 3.0 | 1.0 | 4.2 |
|  | Orthotists and Prosthetists (29-2091) | 7.2 | 8.2 | 16.8 |
|  | Hearing Aid Specialists (29-2092) | 0.0 | 0.0 | 0.0 |
|  | Health Technologists and Technicians, All Other (29-2099) | 2.5 | 2.2 | 3.7 |
|  | Athletic Trainers (29-9091) | **79.5** | 45.5 | 60.5 |
|  | Genetic Counselors (29-9092) | 0.0 | 0.0 | 1.2 |
|  | Surgical Assistants (29-9093) | 0.2 | 0.2 | 8.5 |
|  | Healthcare Practitioners and Technical Workers, All Other (29-9099) | 3.8 | 4.5 | 13.8 |
| Healthcare Support Occupations (31-0000) | Home Health Aides (31-1121) | 6.8 | 2.8 | 5.8 |
|  | Personal Care Aides (31-1122) | 10.2 | 7.5 | 15.2 |
|  | Nursing Assistants (31-1131) | 21.0 | 17.8 | 18.5 |
|  | Orderlies (31-1132) | 26.2 | 25.8 | 16.8 |
|  | Psychiatric Aides (31-1133) | 44.8 | 15.0 | 12.8 |
|  | Occupational Therapy Assistants (31-2011) | 7.2 | 7.5 | 3.8 |
|  | Occupational Therapy Aides (31-2012) | 26.8 | 21.2 | 10.8 |
|  | Physical Therapist Assistants (31-2021) | 2.0 | 0.0 | 0.5 |
|  | Physical Therapist Aides (31-2022) | 0.2 | 0.2 | 1.8 |
|  | Massage Therapists (31-9011) | 5.0 | 9.0 | 10.5 |
|  | Dental Assistants (31-9091) | 0.0 | 3.0 | 12.0 |
|  | Medical Assistants (31-9092) | 0.0 | 0.0 | 0.2 |
|  | Medical Equipment Preparers (31-9093) | 0.0 | 2.2 | 0.8 |
|  | Medical Transcriptionists (31-9094) | 5.8 | 2.0 | 0.0 |
|  | Pharmacy Aides (31-9095) | 9.8 | 9.0 | 9.5 |
|  | Veterinary Assistants and Laboratory Animal Caretakers (31-9096) | 41.5 | 27.5 | 23.0 |
|  | Phlebotomists (31-9097) | 6.8 | 3.5 | 13.8 |
|  | Healthcare Support Workers, All Other (31-9099) | 0.8 | 0.0 | 0.4 |
| Protective Service Occupations (33-0000) | First-Line Supervisors of Correctional Officers (33-1011) | 64.8 | 23.2 | 50.5 |
|  | First-Line Supervisors of Police and Detectives (33-1012) | **83.0** | 44.5 | 53.2 |
|  | First-Line Supervisors of Firefighting and Prevention Workers (33-1021) | **88.5** | 50.0 | **75.2** |
|  | Firefighters (33-2011) | **86.8** | 58.0 | 66.0 |
|  | Fire Inspectors and Investigators (33-2021) | **83.2** | 50.8 | 63.2 |
|  | Forest Fire Inspectors and Prevention Specialists (33-2022) | **90.8** | 55.8 | **87.5** |
|  | Bailiffs (33-3011) | 27.8 | 10.2 | 24.5 |
|  | Correctional Officers and Jailers (33-3012) | 46.8 | 22.5 | 33.2 |
|  | Detectives and Criminal Investigators (33-3021) | 55.7 | 43.1 | 50.2 |
|  | Fish and Game Wardens (33-3031) | **96.8** | 58.8 | 58.5 |
|  | Parking Enforcement Workers (33-3041) | **99.5** | 39.2 | 18.5 |
|  | Police and Sheriff's Patrol Officers (33-3051) | **81.9** | 49.2 | 59.8 |
|  | Transit and Railroad Police (33-3052) | **93.2** | 28.0 | **78.2** |
|  | Animal Control Workers (33-9011) | **93.5** | 40.2 | 44.5 |
|  | Private Detectives and Investigators (33-9021) | 43.0 | 23.5 | 30.8 |
|  | Gambling Surveillance Officers and Gambling Investigators (33-9031) | 16.2 | 10.8 | 15.5 |
|  | Security Guards (33-9032) | 57.5 | 10.0 | 9.2 |
|  | Crossing Guards and Flaggers (33-9091) | **99.8** | 15.8 | 46.8 |
|  | Lifeguards, Ski Patrol, and Other Recreational Protective Service Workers (33-9092) | 67.2 | 52.0 | 60.0 |
|  | Transportation Security Screeners (33-9093) | 22.2 | 26.0 | 45.2 |
|  | Protective Service Workers, All Other (33-9099) | 12.5 | 6.2 | 9.0 |
| Food Preparation and Serving Related Occupations (35-0000) | Chefs and Head Cooks (35-1011) | 36.2 | 32.8 | 51.8 |
|  | First-Line Supervisors of Food Preparation and Serving Workers (35-1012) | 35.0 | 11.2 | 27.8 |
|  | Cooks, Fast Food (35-2011) | 1.2 | 0.0 | 18.2 |
|  | Cooks, Institution and Cafeteria (35-2012) | 2.2 | 6.8 | 13.8 |
|  | Cooks, Private Household (35-2013) | 18.2 | 20.2 | 22.0 |
|  | Cooks, Restaurant (35-2014) | 16.5 | 11.8 | 24.5 |
|  | Cooks, Short Order (35-2015) | 23.0 | 8.8 | 34.8 |
|  | Food Preparation Workers (35-2021) | 17.5 | 15.2 | 6.5 |
|  | Bartenders (35-3011) | 4.8 | 4.5 | 10.8 |
|  | Fast Food and Counter Workers (35-3023) | 6.5 | 4.5 | 10.6 |
|  | Waiters and Waitresses (35-3031) | 9.2 | 22.0 | 7.5 |
|  | Food Servers, Nonrestaurant (35-3041) | 9.5 | 8.8 | 13.0 |
|  | Dining Room and Cafeteria Attendants and Bartender Helpers (35-9011) | 7.8 | 6.0 | 5.0 |
|  | Dishwashers (35-9021) | 40.0 | 34.2 | 12.5 |
|  | Hosts and Hostesses, Restaurant, Lounge, and Coffee Shop (35-9031) | 18.8 | 21.2 | 13.0 |
| Building and Grounds Cleaning and Maintenance Occupations (37-0000) | First-Line Supervisors of Housekeeping and Janitorial Workers (37-1011) | 34.5 | 15.8 | 34.8 |
|  | First-Line Supervisors of Landscaping, Lawn Service, and Groundskeeping Workers (37-1012) | **98.8** | 52.2 | 61.0 |
|  | Janitors and Cleaners, Except Maids and Housekeeping Cleaners (37-2011) | 42.0 | 17.8 | 29.5 |
|  | Maids and Housekeeping Cleaners (37-2012) | 7.8 | 9.5 | 7.8 |
|  | Pest Control Workers (37-2021) | **91.8** | 47.0 | 54.5 |
|  | Landscaping and Groundskeeping Workers (37-3011) | **99.5** | 16.2 | 25.2 |
|  | Pesticide Handlers, Sprayers, and Applicators, Vegetation (37-3012) | **94.0** | 7.2 | 54.8 |
|  | Tree Trimmers and Pruners (37-3013) | **97.8** | 20.8 | 28.5 |
| Personal Care and Service Occupations (39-0000) | First-Line Supervisors of Gambling Services Workers (39-1013) | 6.2 | 15.8 | 9.0 |
|  | First-Line Supervisors of Personal Service Workers (39-1022) | 44.5 | 8.2 | 36.2 |
|  | Animal Trainers (39-2011) | **79.8** | 62.5 | 53.8 |
|  | Animal Caretakers (39-2021) | **75.8** | 41.5 | 46.5 |
|  | Gambling Dealers (39-3011) | 0.0 | 0.2 | 2.5 |
|  | Gambling and Sports Book Writers and Runners (39-3012) | 0.0 | 0.0 | 8.2 |
|  | Motion Picture Projectionists (39-3021) | 20.2 | 4.2 | 9.8 |
|  | Ushers, Lobby Attendants, and Ticket Takers (39-3031) | 15.8 | 7.0 | 10.0 |
|  | Amusement and Recreation Attendants (39-3091) | 69.5 | 58.2 | 54.2 |
|  | Costume Attendants (39-3092) | 11.5 | 9.8 | 27.8 |
|  | Locker Room, Coatroom, and Dressing Room Attendants (39-3093) | 16.2 | 7.5 | 11.8 |
|  | Embalmers (39-4011) | 67.5 | 42.2 | 42.5 |
|  | Funeral Attendants (39-4021) | 55.8 | 25.0 | 33.0 |
|  | Morticians, Undertakers, and Funeral Arrangers (39-4031) | 62.2 | 36.5 | 37.0 |
|  | Barbers (39-5011) | 0.0 | 0.0 | 1.2 |
|  | Hairdressers, Hairstylists, and Cosmetologists (39-5012) | 0.0 | 0.0 | 5.0 |
|  | Makeup Artists, Theatrical and Performance (39-5091) | 58.8 | 56.5 | 54.2 |
|  | Manicurists and Pedicurists (39-5092) | 0.0 | 0.0 | 0.0 |
|  | Shampooers (39-5093) | 8.0 | 4.5 | 12.8 |
|  | Skincare Specialists (39-5094) | 0.2 | 0.2 | 0.0 |
|  | Baggage Porters and Bellhops (39-6011) | **94.5** | 62.0 | 39.2 |
|  | Concierges (39-6012) | 9.5 | 10.2 | 8.2 |
|  | Tour Guides and Escorts (39-7011) | 44.0 | 29.8 | 27.2 |
|  | Travel Guides (39-7012) | 55.0 | 35.0 | 35.2 |
|  | Childcare Workers (39-9011) | 52.0 | 36.0 | 10.2 |
|  | Exercise Trainers and Group Fitness Instructors (39-9031) | 28.5 | 23.8 | 26.2 |
|  | Recreation Workers (39-9032) | 29.5 | 18.8 | 21.2 |
|  | Residential Advisors (39-9041) | 46.5 | 29.8 | 14.8 |
| Sales and Related Occupations (41-0000) | First-Line Supervisors of Retail Sales Workers (41-1011) | 26.2 | 9.5 | 19.2 |
|  | First-Line Supervisors of Non-Retail Sales Workers (41-1012) | 46.8 | 33.2 | 34.2 |
|  | Cashiers (41-2011) | 30.2 | 23.0 | 15.2 |
|  | Gambling Change Persons and Booth Cashiers (41-2012) | 9.0 | 9.2 | 3.5 |
|  | Counter and Rental Clerks (41-2021) | 45.8 | 24.0 | 21.2 |
|  | Parts Salespersons (41-2022) | 57.5 | 25.8 | **84.5** |
|  | Retail Salespersons (41-2031) | 39.5 | 17.5 | 15.2 |
|  | Advertising Sales Agents (41-3011) | 43.5 | 16.0 | 23.2 |
|  | Insurance Sales Agents (41-3021) | 33.8 | 16.0 | 26.5 |
|  | Securities, Commodities, and Financial Services Sales Agents (41-3031) | 4.8 | 6.5 | 14.2 |
|  | Travel Agents (41-3041) | 1.5 | 0.0 | 1.5 |
|  | Sales Representatives, Wholesale and Manufacturing, Technical and Scientific Products (41-4011) | 35.6 | 14.5 | 40.2 |
|  | Sales Representatives, Wholesale and Manufacturing, Except Technical and Scientific Products (41-4012) | 43.5 | 4.2 | 17.2 |
|  | Demonstrators and Product Promoters (41-9011) | 8.0 | 10.2 | 6.0 |
|  | Models (41-9012) | 9.5 | 11.8 | 11.0 |
|  | Real Estate Brokers (41-9021) | 71.8 | 25.2 | 31.8 |
|  | Real Estate Sales Agents (41-9022) | 58.0 | 48.8 | 32.2 |
|  | Sales Engineers (41-9031) | 4.5 | 10.0 | 14.2 |
|  | Telemarketers (41-9041) | 7.0 | 2.2 | 5.2 |
|  | Door-to-Door Sales Workers, News and Street Vendors, and Related Workers (41-9091) | 15.5 | 19.8 | 20.8 |
| Office and Administrative Support Occupations (43-0000) | First-Line Supervisors of Office and Administrative Support Workers (43-1011) | 5.2 | 2.2 | 8.8 |
|  | Switchboard Operators, Including Answering Service (43-2011) | 7.5 | 1.2 | 5.5 |
|  | Telephone Operators (43-2021) | 0.0 | 2.0 | 0.5 |
|  | Bill and Account Collectors (43-3011) | 4.2 | 0.0 | 2.2 |
|  | Billing and Posting Clerks (43-3021) | 6.8 | 1.2 | 0.0 |
|  | Bookkeeping, Accounting, and Auditing Clerks (43-3031) | 10.0 | 2.8 | 8.5 |
|  | Gambling Cage Workers (43-3041) | 7.2 | 0.5 | 6.2 |
|  | Payroll and Timekeeping Clerks (43-3051) | 3.0 | 1.5 | 3.5 |
|  | Procurement Clerks (43-3061) | 8.2 | 7.2 | 28.2 |
|  | Tellers (43-3071) | 5.5 | 4.8 | 4.0 |
|  | Brokerage Clerks (43-4011) | 0.0 | 0.0 | 0.0 |
|  | Correspondence Clerks (43-4021) | 1.2 | 0.0 | 0.0 |
|  | Court, Municipal, and License Clerks (43-4031) | 4.8 | 3.2 | 5.0 |
|  | Credit Authorizers, Checkers, and Clerks (43-4041) | 3.8 | 3.5 | 26.0 |
|  | Customer Service Representatives (43-4051) | 7.2 | 3.0 | 5.0 |
|  | Eligibility Interviewers, Government Programs (43-4061) | 9.5 | 4.0 | 6.5 |
|  | File Clerks (43-4071) | 1.5 | 1.0 | 1.2 |
|  | Hotel, Motel, and Resort Desk Clerks (43-4081) | 10.5 | 6.2 | 4.0 |
|  | Interviewers, Except Eligibility and Loan (43-4111) | 7.0 | 7.0 | 8.2 |
|  | Library Assistants, Clerical (43-4121) | 11.2 | 7.5 | 16.8 |
|  | Loan Interviewers and Clerks (43-4131) | 1.2 | 1.0 | 6.2 |
|  | New Accounts Clerks (43-4141) | 11.5 | 4.0 | 3.5 |
|  | Order Clerks (43-4151) | 16.2 | 0.0 | 14.0 |
|  | Human Resources Assistants, Except Payroll and Timekeeping (43-4161) | 1.5 | 0.5 | 9.5 |
|  | Receptionists and Information Clerks (43-4171) | 0.5 | 0.2 | 3.0 |
|  | Reservation and Transportation Ticket Agents and Travel Clerks (43-4181) | 48.5 | 3.0 | 3.8 |
|  | Cargo and Freight Agents (43-5011) | 19.0 | 12.8 | 23.4 |
|  | Couriers and Messengers (43-5021) | 66.2 | 15.8 | 37.2 |
|  | Public Safety Telecommunicators (43-5031) | 0.8 | 0.8 | 4.0 |
|  | Dispatchers, Except Police, Fire, and Ambulance (43-5032) | 28.0 | 9.8 | 25.8 |
|  | Meter Readers, Utilities (43-5041) | **100.0** | 9.2 | 50.8 |
|  | Postal Service Clerks (43-5051) | 20.0 | 18.8 | 26.8 |
|  | Postal Service Mail Carriers (43-5052) | **97.8** | 32.8 | 49.8 |
|  | Postal Service Mail Sorters, Processors, and Processing Machine Operators (43-5053) | 9.2 | 6.5 | 25.5 |
|  | Production, Planning, and Expediting Clerks (43-5061) | 22.5 | 6.8 | 33.2 |
|  | Shipping, Receiving, and Inventory Clerks (43-5071) | 29.2 | 11.5 | **88.8** |
|  | Weighers, Measurers, Checkers, and Samplers, Recordkeeping (43-5111) | 16.2 | 20.2 | 43.2 |
|  | Executive Secretaries and Executive Administrative Assistants (43-6011) | 3.8 | 3.8 | 3.2 |
|  | Legal Secretaries and Administrative Assistants (43-6012) | 0.5 | 2.0 | 2.5 |
|  | Medical Secretaries and Administrative Assistants (43-6013) | 0.0 | 0.0 | 0.0 |
|  | Secretaries and Administrative Assistants, Except Legal, Medical, and Executive (43-6014) | 9.0 | 1.8 | 1.8 |
|  | Data Entry Keyers (43-9021) | 0.0 | 0.0 | 0.0 |
|  | Word Processors and Typists (43-9022) | 1.5 | 2.2 | 2.0 |
|  | Desktop Publishers (43-9031) | 12.0 | 2.2 | 0.5 |
|  | Insurance Claims and Policy Processing Clerks (43-9041) | 1.0 | 0.2 | 4.8 |
|  | Mail Clerks and Mail Machine Operators, Except Postal Service (43-9051) | 4.0 | 0.2 | 27.2 |
|  | Office Clerks, General (43-9061) | 4.0 | 1.5 | 4.2 |
|  | Office Machine Operators, Except Computer (43-9071) | 18.2 | 6.0 | 9.8 |
|  | Proofreaders and Copy Markers (43-9081) | 5.2 | 2.0 | 3.2 |
|  | Statistical Assistants (43-9111) | 5.5 | 5.5 | 15.0 |
| Farming, Fishing, and Forestry Occupations (45-0000) | First-Line Supervisors of Farming, Fishing, and Forestry Workers (45-1011) | **87.2** | 52.5 | 54.2 |
|  | Agricultural Inspectors (45-2011) | 73.0 | 58.5 | 71.0 |
|  | Animal Breeders (45-2021) | **81.2** | 65.5 | 52.0 |
|  | Graders and Sorters, Agricultural Products (45-2041) | 16.2 | 16.2 | 16.2 |
|  | Agricultural Equipment Operators (45-2091) | **82.5** | 53.0 | 38.5 |
|  | Farmworkers and Laborers, Crop, Nursery, and Greenhouse (45-2092) | **86.2** | 42.5 | 48.8 |
|  | Farmworkers, Farm, Ranch, and Aquacultural Animals (45-2093) | **78.0** | 68.5 | 50.8 |
|  | Fishing and Hunting Workers (45-3031) | **95.2** | 54.8 | 55.5 |
|  | Forest and Conservation Workers (45-4011) | **92.5** | 42.0 | **75.5** |
|  | Fallers (45-4021) | **98.5** | 7.5 | 16.5 |
|  | Logging Equipment Operators (45-4022) | **80.8** | 25.2 | 23.0 |
|  | Log Graders and Scalers (45-4023) | **99.0** | 35.0 | 63.5 |
| Construction and Extraction Occupations (47-0000) | First-Line Supervisors of Construction Trades and Extraction Workers (47-1011) | **85.6** | 45.5 | 53.8 |
|  | Boilermakers (47-2011) | **78.8** | 56.5 | 67.2 |
|  | Brickmasons and Blockmasons (47-2021) | **99.2** | 50.5 | 74.0 |
|  | Stonemasons (47-2022) | **92.2** | 25.2 | 49.2 |
|  | Carpenters (47-2031) | 72.8 | 49.5 | 43.0 |
|  | Carpet Installers (47-2041) | 26.5 | 16.0 | 41.8 |
|  | Floor Layers, Except Carpet, Wood, and Hard Tiles (47-2042) | 19.0 | 11.8 | 57.0 |
|  | Floor Sanders and Finishers (47-2043) | 21.2 | 23.8 | 44.2 |
|  | Tile and Stone Setters (47-2044) | 45.2 | 36.0 | 46.8 |
|  | Cement Masons and Concrete Finishers (47-2051) | **95.2** | 48.0 | 38.2 |
|  | Terrazzo Workers and Finishers (47-2053) | 24.2 | 19.0 | 53.5 |
|  | Construction Laborers (47-2061) | **90.8** | 55.5 | 57.0 |
|  | Paving, Surfacing, and Tamping Equipment Operators (47-2071) | **82.5** | 44.8 | 30.8 |
|  | Pile Driver Operators (47-2072) | **99.5** | 34.2 | 40.8 |
|  | Operating Engineers and Other Construction Equipment Operators (47-2073) | **100.0** | 35.2 | 52.5 |
|  | Drywall and Ceiling Tile Installers (47-2081) | 53.2 | 46.8 | 53.8 |
|  | Tapers (47-2082) | 22.8 | 33.0 | 66.0 |
|  | Electricians (47-2111) | **78.2** | 70.8 | **80.5** |
|  | Glaziers (47-2121) | **86.0** | 69.2 | 71.8 |
|  | Insulation Workers, Floor, Ceiling, and Wall (47-2131) | 72.2 | 59.8 | 72.2 |
|  | Insulation Workers, Mechanical (47-2132) | 57.2 | 41.8 | 73.5 |
|  | Painters, Construction and Maintenance (47-2141) | 59.5 | 27.0 | 43.2 |
|  | Paperhangers (47-2142) | 43.8 | 30.5 | 44.8 |
|  | Pipelayers (47-2151) | **100.0** | 25.0 | 34.0 |
|  | Plumbers, Pipefitters, and Steamfitters (47-2152) | **75.8** | 55.2 | **76.9** |
|  | Plasterers and Stucco Masons (47-2161) | **83.8** | 24.2 | 33.2 |
|  | Reinforcing Iron and Rebar Workers (47-2171) | **90.0** | 30.5 | 47.5 |
|  | Roofers (47-2181) | **99.5** | 30.0 | 70.8 |
|  | Sheet Metal Workers (47-2211) | 37.8 | 35.5 | 64.0 |
|  | Structural Iron and Steel Workers (47-2221) | **95.0** | 57.2 | 61.0 |
|  | Solar Photovoltaic Installers (47-2231) | **92.2** | 68.0 | 73.5 |
|  | Helpers--Brickmasons, Blockmasons, Stonemasons, and Tile and Marble Setters (47-3011) | **83.0** | 46.2 | 57.0 |
|  | Helpers--Carpenters (47-3012) | **79.0** | 49.0 | 61.2 |
|  | Helpers--Electricians (47-3013) | 73.8 | 69.2 | 69.8 |
|  | Helpers--Painters, Paperhangers, Plasterers, and Stucco Masons (47-3014) | 74.0 | 45.8 | 46.8 |
|  | Helpers--Pipelayers, Plumbers, Pipefitters, and Steamfitters (47-3015) | **87.0** | 59.0 | 65.2 |
|  | Helpers--Roofers (47-3016) | **87.5** | 49.2 | 48.2 |
|  | Construction and Building Inspectors (47-4011) | **77.5** | 55.5 | 72.5 |
|  | Elevator and Escalator Installers and Repairers (47-4021) | 56.5 | 39.0 | 70.2 |
|  | Fence Erectors (47-4031) | **94.0** | 44.5 | 38.5 |
|  | Hazardous Materials Removal Workers (47-4041) | 64.5 | 58.0 | 67.2 |
|  | Highway Maintenance Workers (47-4051) | **98.5** | 25.5 | 65.2 |
|  | Rail-Track Laying and Maintenance Equipment Operators (47-4061) | **98.8** | 25.8 | 39.8 |
|  | Septic Tank Servicers and Sewer Pipe Cleaners (47-4071) | **98.8** | 37.2 | 55.0 |
|  | Segmental Pavers (47-4091) | **83.8** | 32.8 | 53.8 |
|  | Construction and Related Workers, All Other (47-4099) | **85.2** | 53.2 | 62.5 |
|  | Derrick Operators, Oil and Gas (47-5011) | **99.8** | 52.8 | 39.5 |
|  | Rotary Drill Operators, Oil and Gas (47-5012) | **99.2** | 67.8 | 57.0 |
|  | Service Unit Operators, Oil and Gas (47-5013) | **100.0** | 52.2 | 54.8 |
|  | Excavating and Loading Machine and Dragline Operators, Surface Mining (47-5022) | **77.5** | 28.8 | 24.0 |
|  | Earth Drillers, Except Oil and Gas (47-5023) | **84.0** | 22.2 | 30.0 |
|  | Explosives Workers, Ordnance Handling Experts, and Blasters (47-5032) | **98.0** | 27.2 | 56.2 |
|  | Continuous Mining Machine Operators (47-5041) | 23.2 | 23.0 | 59.2 |
|  | Roof Bolters, Mining (47-5043) | 32.0 | 32.0 | **87.8** |
|  | Loading and Moving Machine Operators, Underground Mining (47-5044) | 28.0 | 22.8 | 60.8 |
|  | Rock Splitters, Quarry (47-5051) | **94.2** | 36.5 | 38.5 |
|  | Roustabouts, Oil and Gas (47-5071) | **99.5** | 41.2 | **80.2** |
|  | Helpers--Extraction Workers (47-5081) | **91.8** | 34.0 | **89.5** |
| Installation, Maintenance, and Repair Occupations (49-0000) | First-Line Supervisors of Mechanics, Installers, and Repairers (49-1011) | 56.5 | 28.8 | 59.5 |
|  | Computer, Automated Teller, and Office Machine Repairers (49-2011) | 31.2 | 11.0 | 28.0 |
|  | Radio, Cellular, and Tower Equipment Installers and Repairers (49-2021) | 64.0 | 31.0 | 60.2 |
|  | Telecommunications Equipment Installers and Repairers, Except Line Installers (49-2022) | **83.5** | 36.8 | 58.0 |
|  | Avionics Technicians (49-2091) | 42.0 | 28.0 | 52.8 |
|  | Electric Motor, Power Tool, and Related Repairers (49-2092) | 40.5 | 33.5 | **81.2** |
|  | Electrical and Electronics Installers and Repairers, Transportation Equipment (49-2093) | **75.2** | 25.8 | 72.8 |
|  | Electrical and Electronics Repairers, Commercial and Industrial Equipment (49-2094) | 31.2 | 27.2 | 40.0 |
|  | Electrical and Electronics Repairers, Powerhouse, Substation, and Relay (49-2095) | **83.0** | 63.5 | **77.8** |
|  | Electronic Equipment Installers and Repairers, Motor Vehicles (49-2096) | 31.5 | 9.0 | 74.0 |
|  | Audiovisual Equipment Installers and Repairers (49-2097) | 69.5 | 52.8 | 48.8 |
|  | Security and Fire Alarm Systems Installers (49-2098) | 60.8 | 38.0 | 73.0 |
|  | Aircraft Mechanics and Service Technicians (49-3011) | 60.5 | 38.5 | **80.0** |
|  | Automotive Body and Related Repairers (49-3021) | 25.5 | 22.8 | **76.8** |
|  | Automotive Glass Installers and Repairers (49-3022) | **93.8** | 71.5 | **78.2** |
|  | Automotive Service Technicians and Mechanics (49-3023) | 49.8 | 37.2 | 69.2 |
|  | Bus and Truck Mechanics and Diesel Engine Specialists (49-3031) | **82.2** | 49.2 | **88.0** |
|  | Farm Equipment Mechanics and Service Technicians (49-3041) | 74.8 | 56.5 | **90.5** |
|  | Mobile Heavy Equipment Mechanics, Except Engines (49-3042) | **87.2** | 31.5 | **91.0** |
|  | Rail Car Repairers (49-3043) | **81.5** | 45.8 | **85.2** |
|  | Motorboat Mechanics and Service Technicians (49-3051) | **92.0** | 73.2 | **85.8** |
|  | Motorcycle Mechanics (49-3052) | 63.0 | 30.2 | 40.0 |
|  | Outdoor Power Equipment and Other Small Engine Mechanics (49-3053) | 64.5 | 33.2 | **79.5** |
|  | Bicycle Repairers (49-3091) | 17.0 | 17.8 | 25.2 |
|  | Recreational Vehicle Service Technicians (49-3092) | **86.0** | 40.8 | **77.2** |
|  | Tire Repairers and Changers (49-3093) | 47.8 | 43.8 | **90.8** |
|  | Mechanical Door Repairers (49-9011) | **78.5** | 68.0 | 71.8 |
|  | Control and Valve Installers and Repairers, Except Mechanical Door (49-9012) | **90.5** | 54.0 | **75.8** |
|  | Heating, Air Conditioning, and Refrigeration Mechanics and Installers (49-9021) | **76.2** | 53.2 | 73.5 |
|  | Home Appliance Repairers (49-9031) | 62.2 | 47.2 | 67.8 |
|  | Industrial Machinery Mechanics (49-9041) | 42.2 | 27.5 | 73.2 |
|  | Maintenance Workers, Machinery (49-9043) | 47.8 | 33.5 | 67.8 |
|  | Millwrights (49-9044) | **84.0** | 68.2 | **87.5** |
|  | Refractory Materials Repairers, Except Brickmasons (49-9045) | 49.5 | 43.0 | **98.5** |
|  | Electrical Power-Line Installers and Repairers (49-9051) | **100.0** | 22.5 | 73.2 |
|  | Telecommunications Line Installers and Repairers (49-9052) | **95.8** | 64.5 | **75.0** |
|  | Camera and Photographic Equipment Repairers (49-9061) | 3.5 | 3.5 | 20.8 |
|  | Medical Equipment Repairers (49-9062) | 36.5 | 17.8 | 43.2 |
|  | Musical Instrument Repairers and Tuners (49-9063) | 8.5 | 14.8 | 22.5 |
|  | Watch and Clock Repairers (49-9064) | 0.0 | 0.0 | 0.0 |
|  | Maintenance and Repair Workers, General (49-9071) | **81.0** | 32.2 | 69.2 |
|  | Wind Turbine Service Technicians (49-9081) | **93.8** | **86.0** | **94.2** |
|  | Coin, Vending, and Amusement Machine Servicers and Repairers (49-9091) | 57.5 | 41.8 | 49.0 |
|  | Commercial Divers (49-9092) | **90.2** | 58.5 | 66.5 |
|  | Locksmiths and Safe Repairers (49-9094) | **82.2** | 69.0 | 70.2 |
|  | Manufactured Building and Mobile Home Installers (49-9095) | **85.8** | 27.2 | 21.8 |
|  | Riggers (49-9096) | **89.5** | 47.5 | **80.0** |
|  | Signal and Track Switch Repairers (49-9097) | **91.8** | 26.2 | 61.2 |
|  | Helpers--Installation, Maintenance, and Repair Workers (49-9098) | 73.5 | 37.2 | **80.5** |
|  | Installation, Maintenance, and Repair Workers, All Other (49-9099) | **91.8** | 55.8 | **77.5** |
| Production Occupations (51-0000) | First-Line Supervisors of Production and Operating Workers (51-1011) | 40.8 | 33.2 | **76.0** |
|  | Aircraft Structure, Surfaces, Rigging, and Systems Assemblers (51-2011) | 11.2 | 11.0 | 37.2 |
|  | Coil Winders, Tapers, and Finishers (51-2021) | 12.2 | 8.2 | 60.2 |
|  | Electrical and Electronic Equipment Assemblers (51-2022) | 23.0 | 12.5 | 21.8 |
|  | Electromechanical Equipment Assemblers (51-2023) | 2.8 | 0.5 | 5.5 |
|  | Engine and Other Machine Assemblers (51-2031) | 27.5 | 18.8 | 68.2 |
|  | Structural Metal Fabricators and Fitters (51-2041) | 30.0 | 14.8 | **84.0** |
|  | Fiberglass Laminators and Fabricators (51-2051) | 16.8 | 8.5 | 58.0 |
|  | Timing Device Assemblers and Adjusters (51-2061) | 6.5 | 0.5 | 24.2 |
|  | Team Assemblers (51-2092) | 4.8 | 3.0 | 27.5 |
|  | Bakers (51-3011) | 4.0 | 2.0 | 16.2 |
|  | Butchers and Meat Cutters (51-3021) | 7.0 | 1.0 | 11.5 |
|  | Meat, Poultry, and Fish Cutters and Trimmers (51-3022) | 32.2 | 0.0 | 2.0 |
|  | Slaughterers and Meat Packers (51-3023) | 16.8 | 4.0 | 24.2 |
|  | Food and Tobacco Roasting, Baking, and Drying Machine Operators and Tenders (51-3091) | 5.8 | 3.2 | **84.0** |
|  | Food Batchmakers (51-3092) | 0.2 | 0.2 | 34.0 |
|  | Food Cooking Machine Operators and Tenders (51-3093) | 45.5 | 43.5 | 59.2 |
|  | Extruding and Drawing Machine Setters, Operators, and Tenders, Metal and Plastic (51-4021) | 11.2 | 0.2 | 65.0 |
|  | Forging Machine Setters, Operators, and Tenders, Metal and Plastic (51-4022) | 22.5 | 13.8 | 67.2 |
|  | Rolling Machine Setters, Operators, and Tenders, Metal and Plastic (51-4023) | 9.0 | 7.5 | 65.0 |
|  | Cutting, Punching, and Press Machine Setters, Operators, and Tenders, Metal and Plastic (51-4031) | 12.5 | 7.8 | 68.2 |
|  | Drilling and Boring Machine Tool Setters, Operators, and Tenders, Metal and Plastic (51-4032) | 1.8 | 2.5 | 45.0 |
|  | Grinding, Lapping, Polishing, and Buffing Machine Tool Setters, Operators, and Tenders, Metal and Plastic (51-4033) | 13.2 | 9.2 | 38.5 |
|  | Lathe and Turning Machine Tool Setters, Operators, and Tenders, Metal and Plastic (51-4034) | 13.0 | 0.8 | 20.8 |
|  | Milling and Planing Machine Setters, Operators, and Tenders, Metal and Plastic (51-4035) | 6.8 | 2.0 | 64.8 |
|  | Machinists (51-4041) | 8.5 | 8.0 | 20.5 |
|  | Metal-Refining Furnace Operators and Tenders (51-4051) | 20.5 | 6.8 | 73.5 |
|  | Pourers and Casters, Metal (51-4052) | 6.8 | 5.0 | **99.0** |
|  | Model Makers, Metal and Plastic (51-4061) | 1.8 | 0.8 | 46.5 |
|  | Patternmakers, Metal and Plastic (51-4062) | 23.5 | 4.5 | 38.5 |
|  | Foundry Mold and Coremakers (51-4071) | 37.8 | 7.8 | **98.5** |
|  | Molding, Coremaking, and Casting Machine Setters, Operators, and Tenders, Metal and Plastic (51-4072) | 11.0 | 3.5 | 56.8 |
|  | Multiple Machine Tool Setters, Operators, and Tenders, Metal and Plastic (51-4081) | 3.0 | 3.0 | 44.8 |
|  | Tool and Die Makers (51-4111) | 8.0 | 3.5 | 52.5 |
|  | Welders, Cutters, Solderers, and Brazers (51-4121) | 13.0 | 9.8 | 63.2 |
|  | Welding, Soldering, and Brazing Machine Setters, Operators, and Tenders (51-4122) | 17.2 | 14.5 | 73.0 |
|  | Heat Treating Equipment Setters, Operators, and Tenders, Metal and Plastic (51-4191) | 12.8 | 1.0 | **76.8** |
|  | Layout Workers, Metal and Plastic (51-4192) | 30.2 | 35.8 | **86.2** |
|  | Plating Machine Setters, Operators, and Tenders, Metal and Plastic (51-4193) | 11.5 | 6.8 | **78.5** |
|  | Tool Grinders, Filers, and Sharpeners (51-4194) | 22.8 | 0.2 | 67.2 |
|  | Prepress Technicians and Workers (51-5111) | 4.5 | 1.8 | 12.0 |
|  | Printing Press Operators (51-5112) | 3.2 | 0.0 | 4.2 |
|  | Print Binding and Finishing Workers (51-5113) | 2.5 | 1.2 | 21.8 |
|  | Laundry and Dry-Cleaning Workers (51-6011) | 14.8 | 12.2 | 17.2 |
|  | Pressers, Textile, Garment, and Related Materials (51-6021) | 14.5 | 7.2 | 36.8 |
|  | Sewing Machine Operators (51-6031) | 0.0 | 0.0 | 27.8 |
|  | Shoe and Leather Workers and Repairers (51-6041) | 0.0 | 2.2 | 19.2 |
|  | Shoe Machine Operators and Tenders (51-6042) | 0.5 | 0.5 | 53.2 |
|  | Sewers, Hand (51-6051) | 1.2 | 0.0 | 50.0 |
|  | Tailors, Dressmakers, and Custom Sewers (51-6052) | 3.5 | 3.8 | 11.8 |
|  | Textile Bleaching and Dyeing Machine Operators and Tenders (51-6061) | 4.2 | 5.0 | **90.0** |
|  | Textile Cutting Machine Setters, Operators, and Tenders (51-6062) | 10.5 | 1.2 | 68.0 |
|  | Textile Knitting and Weaving Machine Setters, Operators, and Tenders (51-6063) | 0.0 | 0.0 | 31.8 |
|  | Textile Winding, Twisting, and Drawing Out Machine Setters, Operators, and Tenders (51-6064) | 4.5 | 2.2 | 56.0 |
|  | Extruding and Forming Machine Setters, Operators, and Tenders, Synthetic and Glass Fibers (51-6091) | 18.2 | 3.0 | 56.2 |
|  | Fabric and Apparel Patternmakers (51-6092) | 1.2 | 0.0 | 13.8 |
|  | Upholsterers (51-6093) | 5.0 | 5.0 | 39.0 |
|  | Cabinetmakers and Bench Carpenters (51-7011) | 21.5 | 18.5 | **85.5** |
|  | Furniture Finishers (51-7021) | 7.5 | 8.0 | 62.2 |
|  | Model Makers, Wood (51-7031) | 12.0 | 10.2 | **77.0** |
|  | Patternmakers, Wood (51-7032) | 34.8 | 27.8 | 62.8 |
|  | Sawing Machine Setters, Operators, and Tenders, Wood (51-7041) | 26.0 | 20.0 | **87.5** |
|  | Woodworking Machine Setters, Operators, and Tenders, Except Sawing (51-7042) | 17.5 | 9.5 | **92.0** |
|  | Nuclear Power Reactor Operators (51-8011) | 29.0 | 21.2 | 37.8 |
|  | Power Distributors and Dispatchers (51-8012) | 45.0 | 30.8 | 32.0 |
|  | Power Plant Operators (51-8013) | 70.0 | 56.5 | 74.1 |
|  | Stationary Engineers and Boiler Operators (51-8021) | **81.5** | 51.5 | **88.8** |
|  | Water and Wastewater Treatment Plant and System Operators (51-8031) | **75.0** | 55.8 | **79.5** |
|  | Chemical Plant and System Operators (51-8091) | **88.5** | **79.2** | **94.0** |
|  | Gas Plant Operators (51-8092) | **82.2** | 57.5 | 61.0 |
|  | Petroleum Pump System Operators, Refinery Operators, and Gaugers (51-8093) | **96.0** | 72.0 | 64.5 |
|  | Plant and System Operators, All Other (51-8099) | **82.0** | 66.0 | **79.8** |
|  | Chemical Equipment Operators and Tenders (51-9011) | **78.0** | 41.0 | **80.0** |
|  | Separating, Filtering, Clarifying, Precipitating, and Still Machine Setters, Operators, and Tenders (51-9012) | 15.8 | 9.2 | **79.0** |
|  | Crushing, Grinding, and Polishing Machine Setters, Operators, and Tenders (51-9021) | 60.0 | 45.2 | 61.8 |
|  | Grinding and Polishing Workers, Hand (51-9022) | 38.2 | 12.8 | 62.2 |
|  | Mixing and Blending Machine Setters, Operators, and Tenders (51-9023) | 37.2 | 26.5 | 36.0 |
|  | Cutters and Trimmers, Hand (51-9031) | 0.5 | 0.0 | 24.8 |
|  | Cutting and Slicing Machine Setters, Operators, and Tenders (51-9032) | 17.5 | 19.2 | 55.0 |
|  | Extruding, Forming, Pressing, and Compacting Machine Setters, Operators, and Tenders (51-9041) | 3.2 | 4.2 | 58.2 |
|  | Furnace, Kiln, Oven, Drier, and Kettle Operators and Tenders (51-9051) | 19.0 | 23.0 | **86.0** |
|  | Inspectors, Testers, Sorters, Samplers, and Weighers (51-9061) | 23.5 | 16.5 | **85.5** |
|  | Jewelers and Precious Stone and Metal Workers (51-9071) | 2.2 | 2.2 | 10.6 |
|  | Dental Laboratory Technicians (51-9081) | 0.8 | 0.8 | 6.8 |
|  | Medical Appliance Technicians (51-9082) | 4.5 | 0.5 | 7.2 |
|  | Ophthalmic Laboratory Technicians (51-9083) | 0.2 | 0.0 | 9.8 |
|  | Packaging and Filling Machine Operators and Tenders (51-9111) | 18.0 | 5.2 | 40.8 |
|  | Painting, Coating, and Decorating Workers (51-9123) | 5.8 | 6.2 | 60.2 |
|  | Coating, Painting, and Spraying Machine Setters, Operators, and Tenders (51-9124) | 27.8 | 14.2 | 55.5 |
|  | Semiconductor Processing Technicians (51-9141) | 7.0 | 0.2 | 4.0 |
|  | Photographic Process Workers and Processing Machine Operators (51-9151) | 30.8 | 19.0 | 17.2 |
|  | Computer Numerically Controlled Tool Operators (51-9161) | 6.8 | 6.2 | 42.5 |
|  | Computer Numerically Controlled Tool Programmers (51-9162) | 8.2 | 2.8 | 53.0 |
|  | Adhesive Bonding Machine Operators and Tenders (51-9191) | 5.5 | 6.5 | 33.5 |
|  | Cleaning, Washing, and Metal Pickling Equipment Operators and Tenders (51-9192) | 38.2 | 10.8 | 58.8 |
|  | Cooling and Freezing Equipment Operators and Tenders (51-9193) | 55.2 | 31.2 | 63.2 |
|  | Etchers and Engravers (51-9194) | 8.2 | 2.0 | 27.8 |
|  | Molders, Shapers, and Casters, Except Metal and Plastic (51-9195) | 19.2 | 21.1 | 59.9 |
|  | Paper Goods Machine Setters, Operators, and Tenders (51-9196) | 3.0 | 1.5 | 63.5 |
|  | Tire Builders (51-9197) | 20.2 | 9.5 | 58.8 |
|  | Helpers--Production Workers (51-9198) | 28.0 | 15.5 | 68.5 |
| Transportation and Material Moving Occupations (53-0000) | Aircraft Cargo Handling Supervisors (53-1041) | **84.5** | 46.5 | **86.5** |
|  | First-Line Supervisors of Helpers, Laborers, and Material Movers, Hand (53-1042) | 65.1 | 42.1 | 61.0 |
|  | First-Line Supervisors of Material-Moving Machine and Vehicle Operators (53-1043) | 71.2 | 20.5 | 69.8 |
|  | Airline Pilots, Copilots, and Flight Engineers (53-2011) | 60.5 | 15.2 | 34.8 |
|  | Commercial Pilots (53-2012) | 68.0 | 56.0 | 33.5 |
|  | Air Traffic Controllers (53-2021) | 3.2 | 3.5 | 6.0 |
|  | Airfield Operations Specialists (53-2022) | **80.2** | 21.8 | 57.0 |
|  | Flight Attendants (53-2031) | 42.5 | 19.2 | 21.0 |
|  | Ambulance Drivers and Attendants, Except Emergency Medical Technicians (53-3011) | **80.8** | 64.0 | 48.0 |
|  | Driver/Sales Workers (53-3031) | **100.0** | 34.2 | 44.8 |
|  | Heavy and Tractor-Trailer Truck Drivers (53-3032) | **94.0** | 45.2 | 34.5 |
|  | Light Truck Drivers (53-3033) | **78.8** | 39.5 | 45.5 |
|  | Bus Drivers, Transit and Intercity (53-3052) | 54.0 | 27.5 | 15.5 |
|  | Locomotive Engineers (53-4011) | **86.0** | 29.0 | 59.2 |
|  | Rail Yard Engineers, Dinkey Operators, and Hostlers (53-4013) | **92.0** | 24.0 | 67.2 |
|  | Railroad Brake, Signal, and Switch Operators and Locomotive Firers (53-4022) | **93.0** | 24.0 | 53.0 |
|  | Railroad Conductors and Yardmasters (53-4031) | **92.5** | 18.5 | 58.2 |
|  | Subway and Streetcar Operators (53-4041) | **76.0** | 33.0 | 26.5 |
|  | Sailors and Marine Oilers (53-5011) | **100.0** | 21.0 | 36.8 |
|  | Captains, Mates, and Pilots of Water Vessels (53-5021) | **83.5** | 51.0 | 70.2 |
|  | Motorboat Operators (53-5022) | **81.5** | 55.5 | 33.5 |
|  | Ship Engineers (53-5031) | **84.8** | 68.5 | **84.8** |
|  | Bridge and Lock Tenders (53-6011) | 59.2 | 41.2 | 48.8 |
|  | Parking Attendants (53-6021) | 65.2 | 61.2 | 25.0 |
|  | Automotive and Watercraft Service Attendants (53-6031) | **86.2** | 52.2 | **75.0** |
|  | Traffic Technicians (53-6041) | **75.0** | 6.5 | 31.2 |
|  | Transportation Inspectors (53-6051) | 70.1 | 51.2 | 68.5 |
|  | Passenger Attendants (53-6061) | 54.2 | 35.5 | 36.8 |
|  | Conveyor Operators and Tenders (53-7011) | 71.2 | 62.0 | 49.2 |
|  | Crane and Tower Operators (53-7021) | 69.8 | 54.8 | **80.0** |
|  | Dredge Operators (53-7031) | **86.2** | 65.8 | 37.2 |
|  | Hoist and Winch Operators (53-7041) | 51.8 | 33.0 | 67.0 |
|  | Industrial Truck and Tractor Operators (53-7051) | 63.8 | 40.8 | **80.2** |
|  | Cleaners of Vehicles and Equipment (53-7061) | 47.2 | 71.8 | **82.0** |
|  | Laborers and Freight, Stock, and Material Movers, Hand (53-7062) | **82.8** | 69.1 | 62.2 |
|  | Machine Feeders and Offbearers (53-7063) | 18.0 | 11.0 | 42.5 |
|  | Packers and Packagers, Hand (53-7064) | 23.0 | 15.2 | **81.5** |
|  | Stockers and Order Fillers (53-7065) | 18.5 | 10.2 | 32.0 |
|  | Gas Compressor and Gas Pumping Station Operators (53-7071) | **95.8** | **76.0** | 69.2 |
|  | Pump Operators, Except Wellhead Pumpers (53-7072) | **92.2** | 38.8 | 58.5 |
|  | Wellhead Pumpers (53-7073) | **99.2** | 33.0 | 55.5 |
|  | Refuse and Recyclable Material Collectors (53-7081) | **97.2** | 24.8 | 30.8 |
|  | Tank Car, Truck, and Ship Loaders (53-7121) | **94.0** | 48.2 | 72.8 |

^1^ Outdoor heat exposure risk scores were derived from two ONET Work Context Survey questions: “How often does your current job require you to work outdoors, exposed to all weather conditions?” and “How often does your current job require you to work outdoors, under cover (like in an open shed)?” Responses were on a 5-point frequency scale, converted to 0–100 (0 = Never, 25 = Once a year or more but not every month, 50 = Once a month or more but not every week, 75 = Once a week or more but not every day, 100 = Every day); scores ≥75 were classified as elevated risk.
^2^ Indoor heat exposure risk was based on the O*NET question: “How often does your current job require you to work indoors in an environment that is not environmentally controlled (like a warehouse without air conditioning)?” and scored using the same method.
^3^ Approximately 10% (n = 85) of 848 non-military SOC codes lacked ONET data and were excluded. For SOC codes linked to multiple ONET codes, mean exposure scores were used.

### Supplemental Table 3 – Spearman’s correlation between outdoor and indoor occupational heat exposure and social, health, and environmental vulnerability factors in New York State stratified by urbanicity

|  |  | **Total: New York State** | | | **Urban (>75% Urban Population)** | | | **Non-Urban (<75% Urban Population)** | | |
| --- | --- | --- | --- | --- | --- | --- | --- | --- | --- | --- |
| **Domain** | | **Outdoor** | **Indoor** | **Outdoor or Indoor** | **Outdoor** | **Indoor** | **Outdoor or Indoor** | **Outdoor** | **Indoor** | **Outdoor or Indoor** |
|  | **Characteristic** | Spearman's ρ | Spearman's ρ | Spearman's ρ | Spearman's ρ | Spearman's ρ | Spearman's ρ | Spearman's ρ | Spearman's ρ | Spearman's ρ |
| **Social** | |  |  |  |  |  |  |  |  |  |
|  | People of Color Percent | -0.04 | -0.26 | -0.09 | 0.23 | 0.01 | 0.2 | -0.17 | -0.33 | -0.23 |
|  | Individuals Over Age 64 Percent | 0.02 | 0.11 | 0.03 | -0.08 | 0 | -0.08 | -0.04 | -0.07 | -0.06 |
|  | Low Income Percent | 0.21 | 0.15 | 0.24 | 0.23 | 0.17 | 0.27 | 0.41 | 0.4 | 0.45 |
|  | Less than High School Education Percent | 0.28 | 0.12 | 0.28 | 0.37 | 0.21 | 0.37 | 0.46 | 0.38 | 0.49 |
|  | Limited English Speaking Percent | -0.08 | -0.29 | -0.13 | 0.1 | -0.1 | 0.07 | 0.01 | -0.13 | -0.03 |
| **Health** | |  |  |  |  |  |  |  |  |  |
|  | Coronary Heart Disease among Adults - Crude Prevalence | 0.38 | 0.47 | 0.43 | 0.28 | 0.35 | 0.32 | 0.42 | 0.41 | 0.46 |
|  | Chronic Obstructive Pulmonary Disease among Adults - Crude Prevalence | 0.47 | 0.56 | 0.54 | 0.4 | 0.47 | 0.46 | 0.54 | 0.56 | 0.6 |
|  | Current Asthma among Adults - Crude Prevalence | 0.35 | 0.42 | 0.4 | 0.32 | 0.39 | 0.37 | 0.44 | 0.44 | 0.49 |
|  | Diagnosed Diabetes among Adults - Crude Prevalence | 0.41 | 0.31 | 0.42 | 0.45 | 0.35 | 0.46 | 0.48 | 0.42 | 0.51 |
|  | High Blood Pressure among Adults - Crude Prevalence | 0.43 | 0.51 | 0.48 | 0.37 | 0.45 | 0.42 | 0.39 | 0.4 | 0.42 |
| **Environment** | |  |  |  |  |  |  |  |  |  |
|  | Particulate Matter 2.5 | -0.35 | -0.56 | -0.42 | -0.2 | -0.43 | -0.27 | -0.38 | -0.36 | -0.39 |
|  | Ozone | -0.14 | -0.35 | -0.22 | 0.08 | -0.13 | 0.01 | -0.18 | -0.1 | -0.17 |
|  | Nitrogen Dioxide | -0.38 | -0.54 | -0.42 | -0.23 | -0.41 | -0.27 | -0.21 | -0.1 | -0.19 |
|  | Heat Wave - Annualized Frequency | -0.24 | -0.5 | -0.33 | -0.08 | -0.35 | -0.16 | -0.24 | -0.26 | -0.28 |

### Supplemental Table 4– Spearman’s correlation between outdoor and indoor occupational heat exposure and social, health, and environmental vulnerability factors in New Jersey stratified by urbanicity

|  |  | **Total: New Jersey** | | | **Urban (>75% Urban Population)** | | | **Non-Urban (<75% Urban Population)** | | |
| --- | --- | --- | --- | --- | --- | --- | --- | --- | --- | --- |
| **Domain** | | **Domain** | **Outdoor** | **Indoor** | **Outdoor or Indoor** | **Outdoor** | **Indoor** | **Outdoor or Indoor** | **Outdoor** | **Indoor** |
|  | **Characteristic** | Spearman's ρ | Spearman's ρ | Spearman's ρ | Spearman's ρ | Spearman's ρ | Spearman's ρ | Spearman's ρ | Spearman's ρ | Spearman's ρ |
| **Social** | |  |  |  |  |  |  |  |  |  |
|  | People of Color Percent | 0.29 | 0.34 | 0.38 | 0.36 | 0.41 | 0.45 | -0.07 | -0.08 | -0.05 |
|  | Individuals Over Age 64 Percent | -0.14 | -0.17 | -0.19 | -0.18 | -0.21 | -0.22 | 0.17 | 0.14 | 0.18 |
|  | Low Income Percent | 0.56 | 0.54 | 0.61 | 0.59 | 0.57 | 0.63 | 0.52 | 0.53 | 0.58 |
|  | Less than High School Education Percent | 0.62 | 0.63 | 0.68 | 0.65 | 0.66 | 0.7 | 0.56 | 0.55 | 0.61 |
|  | Limited English Speaking Percent | 0.25 | 0.29 | 0.31 | 0.32 | 0.35 | 0.37 | -0.13 | -0.1 | -0.12 |
| **Health** | |  |  |  |  |  |  |  |  |  |
|  | Coronary Heart Disease among Adults - Crude Prevalence | 0.39 | 0.33 | 0.36 | 0.37 | 0.3 | 0.34 | 0.64 | 0.62 | 0.68 |
|  | Chronic Obstructive Pulmonary Disease among Adults - Crude Prevalence | 0.61 | 0.55 | 0.6 | 0.6 | 0.53 | 0.59 | 0.76 | 0.73 | 0.8 |
|  | Current Asthma among Adults - Crude Prevalence | 0.62 | 0.56 | 0.63 | 0.61 | 0.55 | 0.63 | 0.65 | 0.65 | 0.71 |
|  | Diagnosed Diabetes among Adults - Crude Prevalence | 0.59 | 0.58 | 0.64 | 0.6 | 0.6 | 0.64 | 0.64 | 0.63 | 0.69 |
|  | High Blood Pressure among Adults - Crude Prevalence | 0.41 | 0.37 | 0.41 | 0.4 | 0.36 | 0.39 | 0.62 | 0.61 | 0.67 |
| **Environment** | |  |  |  |  |  |  |  |  |  |
|  | Particulate Matter 2.5 | -0.02 | 0.06 | 0.06 | 0.02 | 0.09 | 0.09 | -0.26 | -0.1 | -0.23 |
|  | Ozone | -0.03 | -0.01 | 0.02 | 0.02 | 0.03 | 0.06 | -0.24 | -0.11 | -0.2 |
|  | Nitrogen Dioxide | 0.01 | 0.07 | 0.09 | 0.07 | 0.13 | 0.14 | -0.37 | -0.28 | -0.35 |
|  | Heat Wave - Annualized Frequency | -0.04 | 0.03 | 0.02 | 0 | 0.07 | 0.06 | -0.45 | -0.39 | -0.44 |
